# Intramyocellular triacylglycerol accumulation across weight loss strategies; Sub-study of the CENTRAL trial

**DOI:** 10.1371/journal.pone.0188431

**Published:** 2017-11-30

**Authors:** Yftach Gepner, Ilan Shelef, Dan Schwarzfuchs, Noa Cohen, Nitzan Bril, Michal Rein, Gal Tsaban, Hila Zelicha, Anat Yaskolka Meir, Lilac Tene, Benjamin Sarusy, Philip Rosen, Jay R. Hoffman, Jeffrey R. Stout, Joachim Thiery, Uta Ceglarek, Michael Stumvoll, Matthias Blüher, Meir J. Stampfer, Iris Shai

**Affiliations:** 1 Faculty of Health Sciences, Ben-Gurion University of the Negev, Beer-Sheva, Israel; 2 Institute of Exercise Physiology and Wellness, Sport and Exercise Science; University of Central Florida, Orlando, FL, United States of America; 3 Soroka University Medical Center, Beer-Sheva, Israel; 4 Nuclear Research Center Negev, Dimona, Israel; 5 Department of Medicine, University of Leipzig, Leipzig, Germany; 6 Channing Division of Network Medicine, Department of Medicine, Brigham and Women's Hospital and Harvard School of Public Health, Boston, MA, United States of America; University of Alabama at Birmingham, UNITED STATES

## Abstract

**Background:**

Intramyocellular triacylglycerol (IMTG) is utilized as metabolic fuel during exercise and is linked to insulin resistance, but the long-term effect of weight loss strategies on IMTG among participants with abdominal fat, remain unclear.

**Methods:**

In an 18-month trial, sedentary participants with abdominal fat/dyslipidemia were randomized to either a low-fat (LF) or Mediterranean/low-carbohydrate (MED/LC) diet (including 28g·day^-1^ of walnuts). After 6-months, the participants were re-randomized to moderate intense physical activity (PA+) or non-physical activity (PA-). Magnetic resonance imaging (MRI) was used to quantify changes of IMTG, abdominal sub-depots, hepatic and intermuscular fats.

**Results:**

Across the 277 participants [86% men, age = 48 years, body-mass-index (BMI) = 31kg/m^2^, visceral fat = 33%] 86% completed the 18-m trial. At baseline, women had higher IMTG than men (3.4% vs. 2.3%, p<0.001) and increased IMTG was associated with aging and higher BMI, visceral and intermuscular fats, HbA1c%, HDL-c and leptin(p<0.05), but not with intra-hepatic fat. After 18 month of intervention and a -3 kg mean weight loss, participants significantly increased IMTG by 25%, with a distinct effect in the MED/LC^PA+^ group as compared to the other intervention groups (57% vs. 9.5–18.5%, p<0.05). Changes in IMTG were associated with visceral and intermuscular fat, metabolic syndrome, insulin and leptin (p<0.05 for all), however, these associations did not remain after adjustment for visceral fat changes.

**Conclusions:**

Lifestyle strategies differentially affect IMTG accumulation; combination of exercise with decreased carbohydrate/increased unsaturated fat proportion intake greatly increase IMTG. Our findings suggest that increased IMTG during diet-induced moderate weight loss may not be directly related to cardiometabolic risk.

**Trial registration:**

ClinicalTrials.gov NCT01530724

## Introduction

Intramyocellular triacylglycerol (IMTG) represents ∼1% to 2% of the total fat stores within the body[1] and is used as a substrate source during exercise at low to moderate intensities[1–3]. The accumulation of IMTG is significantly greater in women compared to BMI matched men[4,5]. Furthermore, IMTG has been demonstrated to be correlated with BMI, insulin resistance[6] and with central abdominal fat[7] in sedentary obese subjects who are not physically active. Interestingly, highly trained athletes exhibit similar, if not greater concentrations of IMTG, than obese or type 2 diabetics (“the athlete’s paradox”)[8,9]. Improvements in insulin sensitivity with exercise or calorie restriction and weight loss in sedentary overweight humans is associated with reduction in intra-abdominal fat but not in IMTG[10,11].

IMTG plays an important role as an oxidative substrate during and following physical activity (PA)[2,3]. Some studies have demonstrated that acute post exercise training, IMTG is reduced by 20–30% during the recovery period while muscle glycogen was replenishing[12]. In addition, some studies have reported that high-fat diets (50% to 60%) can increase IMTG content[13–15], while others have reported that a high-fat diet may decease IMTG following low calorie-induced weight loss[16]. Furthermore, combining endurance exercise training with the consumption of a high-fat diet has been shown to increase in IMTG content[17–19]. In addition, other investigations have suggested that saturated fatty acid composition may have a greater effect on IMTG and in the development of skeletal muscle insulin resistance than total fat intake[20,21].

Although weight loss and exercise intervention can both decrease pathogenic fat[22,23] and improve insulin sensitivity[24,25], these interventions may have different effects on IMTG. The physiological importance of IMTG beyond its relationship with abdominal adiposity remains unclear. Furthermore, to our knowledge limited research has examined the chronic effects of weight-loss from different diet and exercise strategies on IMTG. Thus, the purpose of this study was to examine the IMTG response to diets with or without moderate PA, and to assess the association between changes of IMTG with changes in cardiometabolic risk parameters.

## Materials and methods

### Study population

This is a sub-study of the CENTRAL randomized controlled trial (ClinicalTrials.gov identifier: NCT01530724, S1 Table) aimed to assess whether different diet and exercise interventions could preferentially induce the loss of visceral fat in patients with central adiposity (primary endpoint), with changes in other fat depots, including IMTG. The trial involved 277 participants and was conducted between October 2012 and April 2014 at the Nuclear Research Center Negev (Dimona, Israel), a workplace with a dedicated cafeteria and an on-site medical clinic. Inclusion criteria were: abdominal obesity [waist circumference >102cm (40 inches) for men and >88cm (35 inches) for women], or serum triglycerides (TG)>150 mg·dL^-1^ and high-density-lipoprotein cholesterol (HDL-c) <40 mg·dL^-1^ for men and <50 mg·dL^-1^ for women. Exclusion criteria were: serum creatinine ≥ 2mg·dl^-1^; impaired liver function (≥ threefold the upper level of ALT and AST), active cancer, pregnancy or lactation, highly physically active (>3 h·week^-1^) or unable to take part in PA, or participation in another trial. The study protocol was approved by the Medical Ethics Board and the Helsinki Committee of Soroka University Medical Center (S1 Appendix). All participants provided written informed consent and received no financial compensation or gifts.

### Randomization and interventions

**Fig 1:**
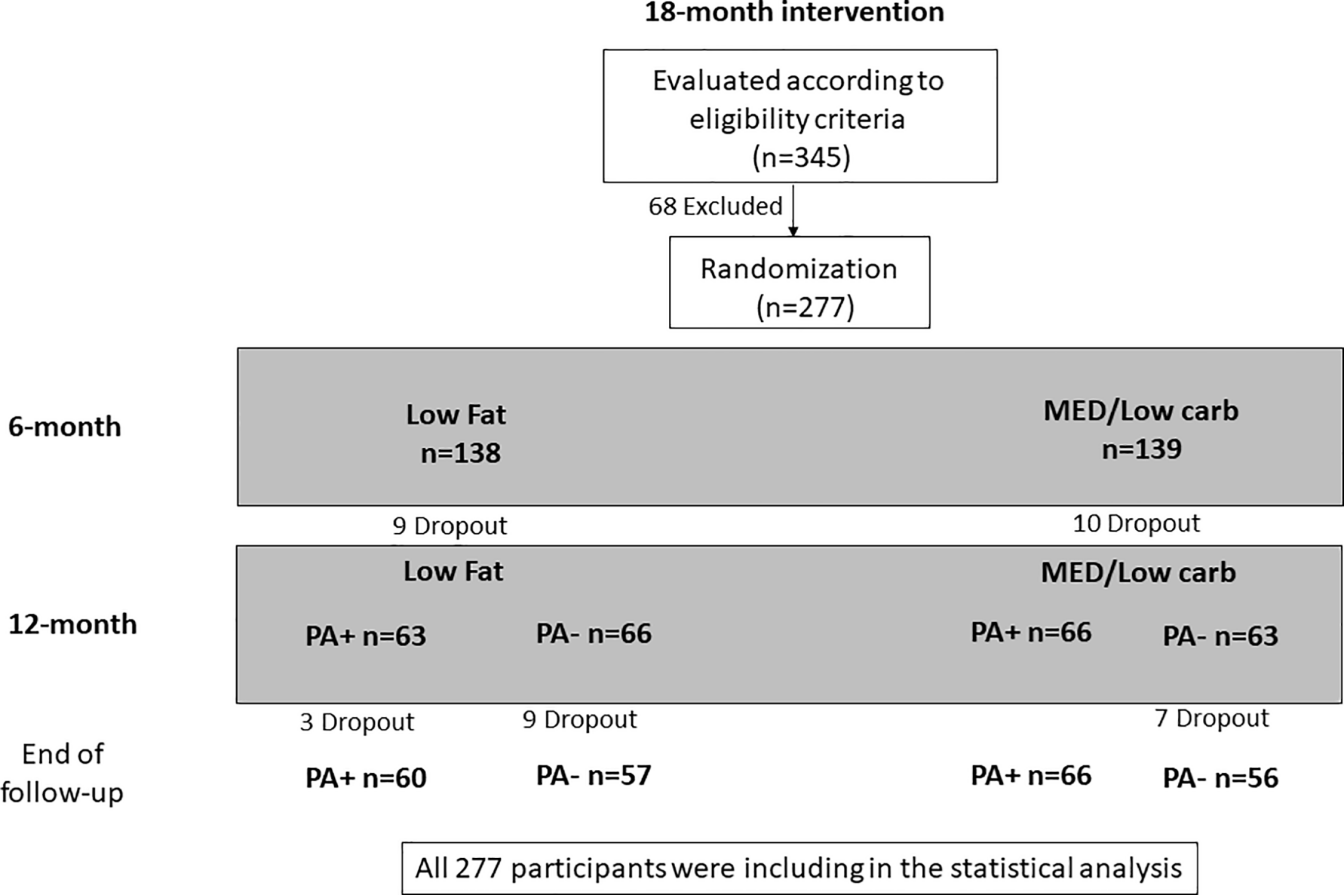
Flow chart of the 18-months study intervention. Intention to treat analysis was performed for all participants. PA+, Physical activity. PA-, Non-Physical activity.

After completion of baseline measures, participants were randomly assigned, without stratification, to one of two equally hypocaloric diets: low-fat diet (LF, n = 138) and Mediterranean/low-carbohydrate/ diet (MED/LC, n = 139). After 6 months of dietary intervention, each diet intervention group was further randomized into PA groups (LF^PA+^, MED/LC^PA+^) or non-PA groups (LF^PA–^, MED/LC^PA–^), (Fig 1).

### Diet intervention

Both diets were aimed at achieving an energy intake of 1500/kcal/day for women and 1800/kcal/day for men, restricting intake of *trans*-fats, refined carbohydrates, and emphasizing the consumption of vegetables. Lunch was provided exclusively by the workplace cafeteria during the work week. A dietitian worked closely with the kitchen staff to adjust the meals to the specific diet groups[26]. The 18-month diet intervention included a 90-minute nutritional session in the workplace with clinical dietitians every week during the first month of intervention, and every month thereafter. To maintain equal intensity of treatment, the workshop format and the quality of the materials were similar across the diet groups, except for instructions and materials specific to each dietary strategy. The LF diet, limited total fat intake to 30% of daily caloric intake, with up to 10% being saturated fat, and no more than 300 mg of cholesterol per day. An additional goal was to increase dietary fiber consumption. Participants were counseled to consume whole grains, vegetables, fruits, and legumes and to limit their consumption of additional fats, sweets, and high-fat snacks. The MED/LC diet combined the Mediterranean and low-carbohydrate diets described in our previous weight loss trial.[26] The diet restricted carbohydrate intake to less than 40 g/day in the first two months (induction phase), and thereafter a gradual increase up to 70 g/day, and increased protein and fat intake, according to the MED diet. The MED/LC diet was rich in vegetables and legumes and low in red meat, with poultry and fish replacing beef and lamb. This group was also provided 28 g of walnuts/day [160 Kcal/84% fat, mostly PUFA (omega-3 α-linolenic acid)] starting from the third month.

### Physical activity intervention

Participants randomized to the PA intervention groups at the 6-month time point received a free supervised gym membership for the following 12 months. The gym was located away from the workplace and the intervention included monthly 60-minute educational workshops, and training group sessions at the gym, directed by a certified fitness instructor, who was blinded to the assigned diets of the participants. The exercise program included three sessions/week of mostly aerobic training. In the first month participants started with 20 minutes of aerobic training at 65% maximum heart rate and 10 minutes of resistance training. Exercise was gradually increased to 45 minutes of aerobic training at 80% of maximum heart rate and 15 minutes of resistance training. The resistance training increased from one set using 60% of the participants’ maximum strength (1RM) to two sets at 80% of the 1RM. Exercises included leg extension, leg curl, elbow flexion, triceps extension, lateral pull-down, lower back extension and bent knee sit-ups. The latter exercise used the participant’s body mass only.

### MRI acquisition and image analysis

All participants underwent whole body MRI imaging. Scans were performed using a 3-Tesla magnet (Intera, Philips, Medical Systems, Netherlands). The MRI scanner utilized a 3D modified DIXON (mDIXON) imaging technique without gaps (2 mm thickness and 2 mm of spacing), a fast-low-angle shot (FLASH) sequence with a multi-echo two-excitation pulse sequence for phase-sensitive encoding of fat and water signals (TR 3.6 ms; TE 1,1.19 ms; TE2 2.3 ms; FOV 520×440×80mm; 2×1.4×1 mm voxel size). Four images of the phantoms were generated, including in-phase, out-phase, fat phase and water phase. A breath-hold technique was used to prevent motion artifacts when the chest and abdomen were scanned. In all simultaneous fat depots quantification and comparisons, observers were blinded to time point and group treatment. We estimated the measurement error by evaluating a phantom included in the MRI acquisition. Utilizing the same software used to assess adipose tissue depot area, the mean ± SEM phantom area was 1.4693±0.0046 cm^2^, corresponding to the < 3% error reported in the literature.[27] All fat depots were assessed by one or two raters. The Inter-observer and intra-observer correlations were > 0.96 (p < 0.001) for all measured fat storage pools, and 0.95, p < 0.001 for pericardial fat volume.

#### Intramyocellular triacylglycerol

The IMTG was assessed by utilizing the region of interest (ROI) technique[28]. This method is based on comparison of tissue density (Fat/Fat+Water) in the selected regions. Using semi-automatic PRIDE software from Philips Medical Systems, we analyzed the middle hip 2D image in the central area of four muscles: rectus femoris, vastus lateralis, adductor magnus and semitendinosus. Mean percentage of IMTG was calculated by using all the values of each ROI.

#### Abdominal fat sub-depots

The quantification of the three sub-depots in the abdomen [superficial subcutaneous adipose tissue (superficial-SAT), deep-SAT and visceral (VAT)] was assessed by using a MATLAB-based program. The MRI scan allows visualizing the fascia superficialis as a fine black line, and to divide superficial-SAT from deep-SAT we drew a continuous line over the fascia superficialis. We selected the specified fat mass area, by mean of three slices L2-L3, L5-L4 and L5-S1, using semiautomatic method software, and quantified the fat mass regions[29,30].

#### Hepatic fat

We quantified the percentage of hepatic fat using PRIDE software (Philips Medical Systems). We calculated mean percentage from four 2D slices (3cm intervals divided into quarters) by utilizing the region of interest (ROI) approach, which is based on measurements of tissue densities (fat/fat+water) using the Fat Ratio Calculation.[31] We divided each slice into quarters, and chose ROIs in each of the four quarters in order to represent the entire liver. We determined the mean percentage of fat for each slice and quarter, and then calculated the mean percentage of fat in the liver as a whole.

#### Femoral intermuscular adipose tissue

Femoral intermuscular fat was quantified from a single 2D fat-phase axial slice from the mid-thigh of the right leg, from the femoral head to the medial and lateral condyle. Our semi-automatic MATLAB-based program was applied to distinguish between adipose and lean tissues and to calculate the area (cm^2^) of femoral intermuscular adipose tissue[32].

### Anthropometric measurements

Height was measured to the nearest millimeter by using a standard wall-mounted stadiometer. Waist circumference (WC) was measured to the nearest millimeter with an anthropometric measuring tape; the measurement was made half-way between the last rib and the iliac crest. Body weight was measured monthly without shoes to the nearest 0.1kg.

### Blood measures

Resting blood samples were obtained prior to each testing session. All blood samples were obtained following a 15-min equilibration period. Each participant’s blood samples were obtained at the same time of day during each session following an overnight fast. Fasting blood samples were stored at -80°C. Fasting plasma glucose (FPG) was measured by Roche GLUC 3 (hexokinase method). Plasma insulin was measured with the use of an enzyme immunometric assay [Immulite automated analyzer, Diagnostic Products, coefficient of variation (CV) = 2.5%]. Serum total cholesterol (CV = 1.3%), high-density-lipoprotein cholesterol (HDL-c), low-density-lipoprotein (LDL) cholesterol, and triglycerides (CV = 2.1%) were determined enzymatically with a Cobas 6000 automatic analyzer (Roche). Plasma leptin levels were assessed by ELISA (Mediagnost), with a CV of 2.4%. All biochemical analyses were performed at the University of Leipzig, Germany.

### Dietary and exercise compliance

Adherence to the diet was assessed via a self-administered validated electronic 127 item food-frequency questionnaire (FFQ).[33,34] Adherence to exercise was followed by an electronic self-reported validated PA questionnaire[35] and by electronically monitoring entry to the gym. Text messages were sent to update participants and to motivate adherence to the diets on specific occasions (such as before and after holidays). By using electronic questionnaires,[26] the completeness of the data was ensured by prompting the participants when a question was not answered or when an answer was not within a logical range. Symptoms, adverse effects, quality of life, and medication usage were also followed electronically.

### Statistical analysis

The primary aim of the main CENTRAL study was change in body fat distribution over 18 months of intervention, and the main primary specific endpoint was VAT. The priori hypothesis was that VAT could be differentially altered by lifestyle intervention strategies. The sample size was estimated based on findings from a previous 14-week intervention study, in which 33 postmenopausal obese women (57yr, 92kg, 36% body fat) were randomized to one of three interventions: diet alone, exercise alone, and diet and exercise group and significant relative change in VAT of 12.8% (P<0.05) was found. Thus, the minimum detectable effect after 18 months for the primary VAT between the intervention groups was estimated as 3.57cm2, and for an alpha = 5% and power = 80%, 250 participants were required (calculated using Winpepi software). We increased our sample size from 250 (planned protocol) to 278 participants in order to reach significant differences in other sub-studies analysis.

The aim of this sub-study was changes in IMTG over 18 months of intervention, and the secondary was the associations between IMTG to cardiometabolic biomarkers. From one MRI scan at baseline we were unable to analyze IMTG, therefore, the sample size for this sub-study was 277 participants. A post-hoc power calculation analysis for this sub-study was based on 0.47% differences in IMTG deltas between the LF^PA-^ and the MED/LC^PA+^ groups with 1.18 and 1.14 standards deviations, yield power of 92.9% between intervention groups (calculated using Winpepi software).

We calculated mean ± standard deviation of IMTG percentages and all adipose tissue. We performed intention-to-treat analysis, including all 277 participants, by imputing the missing observations for all adipose tissues for 38 individuals by the multiple imputation technique, wherein the following predictors were used in the imputation model: age, gender, baseline weight, baseline BMI and waist circumference at the end of the intervention. For missing data of body weight, we used the last observation carried forward. Pearson correlations were used to assess selected bivariate relationships at baseline. To test the effect between intervention groups differences on IMTG changes, we performed multivariate linear regression models, using dummy variables of the intervention strategies and adjusting gender, age and visceral adipose tissue changes. Paired sample t-tests were used to assess changes from baseline to 18-months within each intervention group. To test the association between IMTG and selected cardiometabolic parameters at baseline and over the intervention, we used a linear regression model adjusted for the interventions groups and to VAT changes. Statistical analysis was performed with IBM SPSS version 23. A criterion alpha level of p ≤ 0.05 was used to determine statistical significance.

## Results

### Baseline

Baseline characteristics of the participants across intervention groups are shown in Table 1. The participants (age = 47.9 ± 9.3 y, 86.4% males, BMI = 30.6 ± 3.9) had on average across all four muscles 2.4 ± 1.6% IMTG. Specifically, IMTG in the rectus femoris averaged 0.7 ± 1.2%, in the vastus lateralis 1.7 ± 2.1%, in the adductor magnus 2.8 ± 2.2% and in the semitendinosus 4.5 ± 3.4%. The mean percentage of each abdominal fat tissue compartment in the entire population was: superficial-SAT = 27%, deep-SAT = 40% and VAT = 33%. Females had a greater amount (p < 0.001) of IMTG 3.4 ± 1.9% than men 2.3 ± 1.5%. No statistical differences were found at baseline between the intervention groups for IMTG or any other parameters. At baseline, IMTG was positively associated with age (r = 0.18, p = 0.001), waist circumference (r = 0.17, p = 0.014), BMI (r = 0.18, p = 0.002), HDL-c (r = 0.15, p = 0.014) and area of abdominal sub-adipose tissue: superficial-SAT (r = 0.22, p < 0.001), deep-SAT (r = 0.16, p < 0.001), VAT (r = 0.14, p = 0.007) and IMAT (r = 0.54, p < 0.001). No significant association was observed between IMTG to hepatic fat (r = -0.08, p = 0.15) and metabolic syndrome criteria (r = -0.02, p = 0.77). Only 19% of the participants were regularly taking prescription medications, with similar changes noted during the intervention for all groups.

**Table 1:** Characteristics of the study population across intervention groups.

	Low-Fat PA-	Low-Fat PA+	MED/Low-Carb PA-	MED/Low-Carb PA+	All (n = 277)	P between groups
Age, y	49.5±9.2	47.2±9.0	47.0±8.8	47.8±9.8	47.9±9.3	0.33
Waist circumference, cm	105±9.5	106±8.5	106±11	108.0±8.5	106.0±10	0.51
BMI, kg/h2	31.1±3.9	30.3±3.4	30.9±4.4	30.99±3.3	30.6±3.9	0.68
Blood pressure, mmHg						
Systolic	125±16	122±13	124±18	126±16	124±16	0.49
Diastolic	79±11	78±10	81±12	82±11	80±11	0.18
Adipose depots						
IMTG, %	2.7±1.8	2.3±1.4	2.4±1.5	2.1±1.3	2.4±1.6	0.13
Visceral AT, cm^2^	177±71	181±65	160.5±61	184.3±65	175±66	0.14
Deep SAT, cm^2^	208±67	216±70	219±87	220±70	216±74	0.78
Superficial SAT, cm^2^	144±70	139±51	150±75	133±47	142±63	0.45
Intra Hepatic fat, %	10.8±10	9.2±9.0	10.0±10	10.4±11	10.2±10	0.82
IMAT, cm^2^	10.4±5.4	10.1±4.8	8.74±4.1	9.23±3.5	9.6±4.5	0.093
Blood biomarkers						
HDL cholesterol, mg/dl	43.8±13	42.5±12	42.4±10	43.7±9.3	43.1±12	0.82
LDL cholesterol, mg/dl	123±33	124±29	120±34	121±27	122±43	0.88
Triglyceride, mg/dl	71.7±41	78.7±44	73.5±41	66.4±36	72.6±41	0.41
Glucose, mg/dl	106±17	106±18	107±18	108±23	107±19	0.91
HbA1c, %	5.54±0.5	5.52±0.4	5.54±0.4	5.58±0.5	5.5±0.5	0.91
HOMA-IR	4.42±2.6	4.71±3.4	4.74±3.8	4.48±2.6	4.6±3.2	0.92
Leptin, ng/mL	16.0±16	12.6±6.8	14.7±15	14.4±8.6	14.5±12	0.47

BMI: body mass index; SAT, subcutaneous adipose tissue; IMTG: Intramyocellular triacylglycerol IMAT: intermuscular adipose tissue. Values in the Table are means ± standard deviation.

### Adherence

Following the 18-month intervention the retention rate of participants was 86%, with similar demographic and metabolic profiles between completers and non-completers. At baseline, there were no significant differences in the metabolic equivalent (MET) between the PA groups or in consumption of energy or macronutrients between the LF and MED/LC diet groups. However, during the intervention the PA+ groups significantly increased their MET as compared with the PA–groups (19.0 MET·week^-1^ vs. 2.1 MET·week^-1^; p = 0.009). According to the self-reported FFQ, participants adhered to the dietary guidelines for the group that they were randomized (S1 Fig).

### 18-months changes

**Fig 2:**
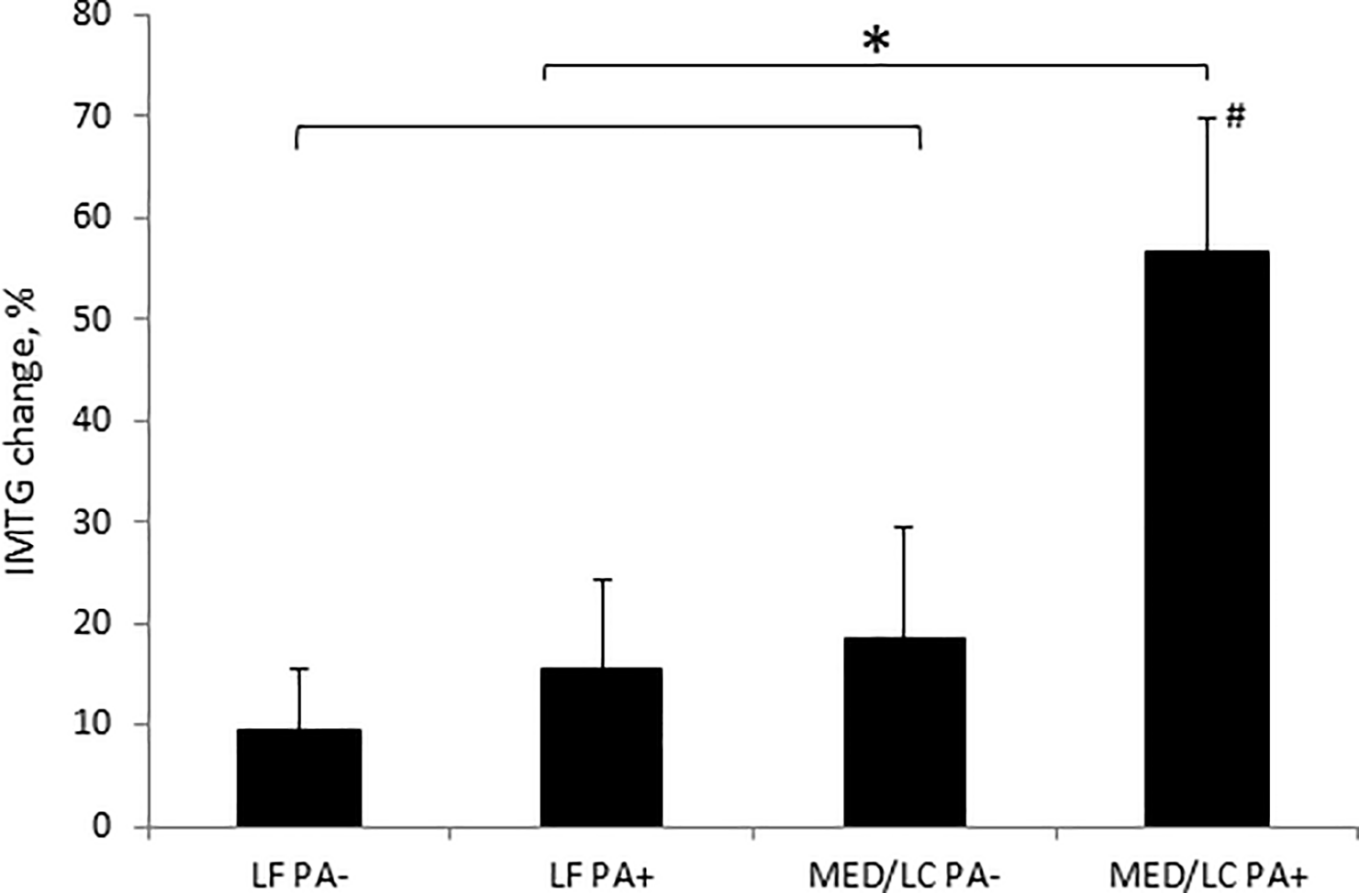
Effect of dietary strategies with or without physical activity on intramyocellular triacylglycerol over 18 months of intervention. Values in the Figure are means ± standard errors. Multivariate linear model adjusted for age, sex and visceral fat changes. *p<0.05, Mediterranean/ Low-carbohydrate diet with physical activity significantly increased intramyocellular triacylglycerol as compared to each of the other intervention groups. # p<0.05, paired t-test was used to test changes over time. LFPA-: Low fat diet non-physical activity; LFPA+: Low fat diet with physical activity; MED/LCPA-: Mediterranean/low-carbohydrate/ diet non-physical activity; MED/LCPA+: Mediterranean/low-carbohydrate/ diet with physical activity.

Following the 18-month intervention a significant decrease was found, for all participants combined, in the change in body weight (-3.0 ± 5.5 kg, p < 0.001) and in insulin-sensitivity parameters, such as HbA1c (-0.05 ± 0.31%, p = 0.022) and HOMA-IR (-0.9 ± 2.4, p < 0.001). IMTG was increased by 0.2% [(95%CI 0.04 to 0.35), relative change 25%, p = 0.008] for the entire study population. Upon further examination, elevations in IMTG was significant only in the MED/LC^PA+^ group [0.56% (95%CI 0.21 to 0.91), relative change 56%, p = 0.002], while no significant changes were found in the other intervention groups [(ranges = 0.03–0.12%, relative change range 9.7% to 18.5%, p > 0.39), (Fig 2). The change observed in the MED/LC^PA+^ combination group was significantly greater than each of the other groups (p = 0.001) in multivariate model adjusted for age, sex and 18-month visceral fat changes, (Fig 2). Similarly, we found that the MED/LC diet further increased IMTG by 24% (95% CI = 4.82 to 43.87) than the LF diet, and the PA+ groups increased IMTG by 25% (95% CI = 4.34 to 44.6) compared to the PA- groups.

### Association with body fat, metabolic syndrome and selected biomarkers

The association between changes of IMTG, body fat, metabolic syndrome and selected biomarkers changes can be observed in Table 2. In controlling for intervention groups, an increase of IMTG was associated with an increase in IMAT (β = 0.572, p < 0.001), intra-abdominal obesity (β = 0.167, p = 0.013), %HbA1c (β = 0.151, p = 0.027), total cholesterol (β = 0.179, p = 0.009) and LDL-c (β = 0.194, p = 0.009). However, when the model was further adjusted for VAT changes, the associations between IMTG and the obesity parameters, abdominal fat or lipid/glycemic biomarkers were not maintained, but only with IMAT and LDL-c.

**Table 2:** Associations between 18-month changes of intramyocellular triacylglycerol and body fat, metabolic syndrome and selected biomarkers.

	Adjusted for intervention group		Adjusted for intervention group and 18m visceral fat changes	
	Δβ	p	β	p
18-month changes				
Δ Visceral fat	0.167	0.013	——-	——-
Δ IMAT	0.572	<0.001	0.0.542	<0.001
Δ Intra Hepatic fat	0.113	0.099	0.042	0.500
Δ Metabolic syndrome parameters	-0.058	0.398	0.025	0.528
Blood biomarkers				
Δ Cholesterol	0.179	0.009	0.122	0.072
Δ LDL-c	0.194	0.005	0.155	0.025
Δ HDL-c	0.017	0.809	0.102	0.125
Δ Triglyceride	0.110	0.111	0.003	0.960
Δ Insulin	0.143	0.039	0.108	0.125
Δ HbA1c	0.151	0.027	0.102	0.110
Δ HOMA-IR	0.109	0.117	0.075	0.291
Δ Leptin	0.020	0.774	-0.030	0.673

Linear regression adjusted for the 4 intervention groups and further for visceral fat changes. The amount and direction by which changes in IMTG (intramyocellular triacylglycerol) was associated with changes in selected parameters represented by β standardized coefficient. IMAT: intermuscular adipose tissue.

## Discussion

In this 18-month randomized control trial we assessed the long-term effect of weight loss strategies combined with moderate exercise on the dynamics of IMTG changes in 277 overweight or dyslipidemia participants. During moderate weight loss, IMTG increased compared to baseline and particularly in the MED/LC diet combined with PA. The increase in IMTG was not, however, independently associated with changes in cardiometabolic markers. Increasing the IMTG accumulation with a healthy lifestyle intervention, therefore, has more to do with energy substrate supply during times of metabolic need rather than being related to cardiometabolic risk.

There are several limitations within the current study. The exercise intensity and type of exercise (aerobic versus resistance) assessment was limited, although electronic gym entry records and the PA questioner, the ability to verify exercise adherence was limited. The reliance of self-reported dietary intake is a limitation based on the assumption that the participants were accurate and honest. Regardless, the accuracy of the self-reported dietary intake has been previously validated[36]. Another limitation is that the free fat mass and total fat mass measurements were not measured, and thus was unable to test the independent relationship between IMTG changes and cardiometabolic markers beyond total body weight, but only in adjusted for VAT changes. The small sample size of women, reflecting the workplace gender profile, limits our ability to apply results for women. The strength of the study is highlighted by the long-term intervention, the large sample size, the high rate of adherence in an isolated workplace, the used of MRI measurement, and the parallel study design in which all participants started the interventions simultaneously.

The most important finding of this study was the differential effect between the intervention strategies on changes in IMTG. Specifically, the MED/LC diet and the PA+ group appeared to induce the greatest increase in IMTG. These results are consistent with previous studies[17,37], and may be explained by increases in mitochondrial density and intrinsic mitochondrial function in response to prolonged exercise training[38,39]. Nevertheless, the apparent increase in IMTG accumulation in the exercise activity group may represent one of the many metabolic adaptations related to short term endurance training[3,40,41], similar to the increases reported in muscle glycogen storage and mitochondrial density[5,42].

In the current trial, the MED/LC diet group experienced a 24% greater increase in IMTG than the LF diet, despite a similar decrease in body mass. It has been reported that high‐fat diets may increase IMTG accumulation in both healthy[13,43] and athletics[14] individuals, with high inter‐individual variation of IMTG trajectory after fat loading. Thus, IMTG accumulation may be partly involved in the mechanism of lipid content by the either quality or quantity of fat intake in the diet. Therefore, increases in IMTG following consuming a healthy hypocaloric diet, including increases in unsaturated fat (mostly PUFA) and decreases in saturated fat and carbohydrate, may explain the lack of an association between excessive fat accumulation in skeletal muscle and cardiometabolic risk markers. These findings are consistent with previous work from our team[26,44] and others[45] that reported on the cardiometabolic benefits of the MED/LC diet as compared to a LF diet.

Several studies have reported on the relationship between IMTG and insulin resistance[2,8,46]. In this study we found a significant reduction in insulin-sensitivity. Moreover, the significant increase in IMTG was associated with %HbA1c and HOMA-IR, but when VAT changes was further adjusted, the relationship was no longer maintained. Therefore, our results may provide additional evidence on the athletes’ paradox’, in which lifestyle modification in overweight sedentary individuals, particularly with exercise training[47,48], can improve insulin resistance, as estimated by % HbA1c and HOMA-IR, despite having higher IMTG content.

In summary, this investigation appears to be the first a long-term trial demonstrating that IMTG is differentially affected by lifestyle strategies. Interestingly, the PA or MED/LC diet promoted resulted in significant increases in IMTG accumulation, however, the combination of the two induced the greatest effect. The change of IMTG content did not alter the result in an independent association with cardiometabolic markers, suggesting that increased in IMTG during healthy lifestyle intervention may be a desirable metabolic adaptation.

## Supporting information

S1 Fig Changes in food group intake after 6 and 18 months of intervention across diet groups. Each bar in the figure represents the mean changes and direction of the food group as compared to baseline. T-test was used to assess differences between diet groups, *P < 0.05.
(TIF)

S1 Table CONSORT checklist. (DOC)

S1 Appendix Study protocol. (DOCX)
