# Supplementary figures and images for: Intramyocellular triacylglycerol accumulation across weight loss strategies; Sub-study of the CENTRAL trial
Source: PLoS One. 2017 Nov 30;12(11):e0188431. doi: 10.1371/journal.pone.0188431 (PMC5708655; doi:10.1371/journal.pone.0188431)

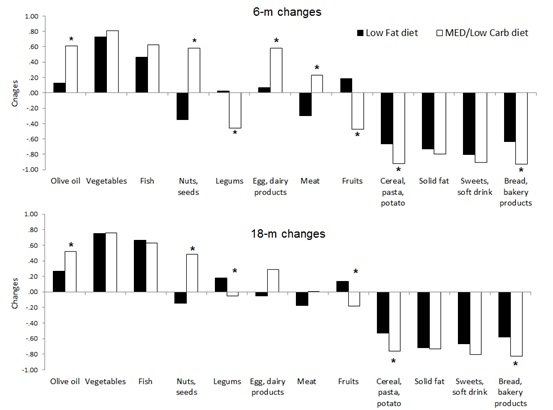

Supplement: S1 Fig — Each bar in the figure represents the mean changes and direction of the food group as compared to baseline. T-test was used to assess differences between diet groups, *P < 0.05. (TIF) [file pone.0188431.s001.tif]
