# Supplementary material for: Intramyocellular triacylglycerol accumulation across weight loss strategies; Sub-study of the CENTRAL trial
Source: PLoS One. 2017 Nov 30;12(11):e0188431. doi: 10.1371/journal.pone.0188431 (PMC5708655; doi:10.1371/journal.pone.0188431)
Supplement: S1 Appendix — (DOCX) [file pone.0188431.s003.docx]

**Differential effect of weight-loss diet strategies on dynamics of abdominal and other fat depots and metabolic risk – the CENTRAL study: intervention trial**

**Protocol Identifying Number: 256/09**

**Principal Investigator: Dr. Ilan Shelef, MD, Soroka Medical Center**

**Funded by: Israel Science Foundation (ISF)**

**Draft or Version Number: v.1.0**

**February, 2012**

Table of Contents

[LIST OF ABBREVIATIONS 1](#_Toc444769362)

[STATEMENT OF COMPLIANCE 1](#_Toc444769363)

[PROTOCOL SUMMARY 1](#_Toc444769364)

[SCHEMATIC OF STUDY DESIGN 1](#_Toc444769365)

[1 KEY ROLES 1](#_Toc444769366)

[2 INTRODUCTION: BACKGROUND INFORMATION AND SCIENTIFIC RATIONALE 1](#_Toc444769367)

[2.1 Background Information 1](#_Toc444769368)

[2.2 Rationale 2](#_Toc444769369)

[2.3 Potential Risks and Benefits 2](#_Toc444769370)

[2.3.1 Known Potential Risks 2](#_Toc444769371)

[2.3.2 Known Potential Benefits 2](#_Toc444769372)

[3 OBJECTIVES AND PURPOSE 2](#_Toc444769373)

[4 STUDY DESIGN AND ENDPOINTS 2](#_Toc444769374)

[4.1 Description of the Study Design 2](#_Toc444769375)

[4.2.1 Primary Endpoint 2](#_Toc444769376)

[4.2.2 Secondary Endpoints 2](#_Toc444769377)

[4.2.3 Exploratory Endpoints 2](#_Toc444769378)

[5 STUDY ENROLLMENT AND WITHDRAWAL 2](#_Toc444769379)

[5.1 Participant Inclusion Criteria 2](#_Toc444769380)

[5.2 Participant Exclusion Criteria 2](#_Toc444769381)

[5.3 Strategies for Recruitment and Retention 2](#_Toc444769382)

[5.4 Participant Withdrawal or Termination 3](#_Toc444769383)

[5.4.1 Reasons for Withdrawal or Termination 3](#_Toc444769384)

[5.4.2 Handling of Participant Withdrawals or Termination 3](#_Toc444769385)

[5.5 Premature Termination or Suspension of Study 3](#_Toc444769386)

[6 STUDY AGENT 3](#_Toc444769387)

[6.1 Study Agent(s) and Control Description 3](#_Toc444769388)

[6.1.1 Acquisition 3](#_Toc444769389)

[6.1.2 Formulation, Appearance, Packaging, and Labeling 3](#_Toc444769390)

[6.1.3 Product Storage and Stability 3](#_Toc444769391)

[6.1.4 Preparation 3](#_Toc444769392)

[6.1.5 Dosing and Administration 3](#_Toc444769393)

[6.1.6 Route of Administration 3](#_Toc444769394)

[6.1.7 Starting Dose and Dose Escalation Schedule 3](#_Toc444769395)

[6.1.8 Dose Adjustments/Modifications/Delays 3](#_Toc444769396)

[6.1.9 Duration of Therapy 4](#_Toc444769397)

[6.1.10 Tracking of Dose 4](#_Toc444769398)

[6.1.11 Device Specific Considerations 4](#_Toc444769399)

[6.2 Study agent Accountability Procedures 4](#_Toc444769400)

[7 STUDY PROCEDURES AND SCHEDULE 4](#_Toc444769401)

[7.1 Study Procedures/Evaluations 4](#_Toc444769402)

[7.1.1 Study specific procedures 4](#_Toc444769403)

[7.1.2 Standard of care study procedures 4](#_Toc444769404)

[7.2 Laboratory Procedures/Evaluations 4](#_Toc444769405)

[7.2.1 Clinical Laboratory Evaluations 4](#_Toc444769406)

[7.2.2 Other Assays or Procedures 4](#_Toc444769407)

[7.2.3 Specimen Preparation, Handling, and Storage 4](#_Toc444769408)

[7.2.4 Specimen Shipment 4](#_Toc444769409)

[7.3 Study Schedule 4](#_Toc444769410)

[7.3.1 Screening 5](#_Toc444769411)

[7.3.2 Enrollment/Baseline 5](#_Toc444769412)

[7.3.3 Follow-up 5](#_Toc444769413)

[7.3.4 Final Study Visit 5](#_Toc444769414)

[7.3.5 Early Termination Visit 5](#_Toc444769415)

[7.3.7 Schedule of Events Table 5](#_Toc444769416)

[7.4 Justification for Sensitive Procedures 5](#_Toc444769417)

[7.5 Concomitant Medications, Treatments, and Procedures 5](#_Toc444769418)

[7.5.1 Precautionary Medications, Treatments, and Procedures 5](#_Toc444769419)

[7.6 Prohibited Medications, Treatments, and Procedures 5](#_Toc444769420)

[7.7 Prophylactic Medications, Treatments, and Procedures 6](#_Toc444769421)

[7.8 Rescue Medications, Treatments, and Procedures 6](#_Toc444769422)

[7.9 Participant Access to Study Agent At Study Closure 6](#_Toc444769423)

[8 ASSESSMENT OF SAFETY 6](#_Toc444769424)

[8.1 Specification of Safety Parameters 6](#_Toc444769425)

[8.1.1 Definition of Adverse Events (AE) 6](#_Toc444769426)

[8.1.2 Definition of Serious Adverse Events (SAE) 6](#_Toc444769427)

[8.1.3 Definition of Unanticipated Problems (UP) 6](#_Toc444769428)

[8.2 Classification of an Adverse Event 6](#_Toc444769429)

[8.2.1 Severity of Event 6](#_Toc444769430)

[8.2.2 Relationship to Study Agent 6](#_Toc444769431)

[8.2.3 Expectedness 6](#_Toc444769432)

[8.3 Time Period and Frequency for Event Assessment and Follow-Up 6](#_Toc444769433)

[8.4 Reporting Procedures 7](#_Toc444769434)

[8.4.1 Adverse Event Reporting 7](#_Toc444769435)

[8.4.2 Serious Adverse Event Reporting 7](#_Toc444769436)

[8.4.3 Unanticipated Problem Reporting 7](#_Toc444769437)

[8.4.4 Events of Special Interest 7](#_Toc444769438)

[8.4.5 Reporting of Pregnancy 7](#_Toc444769439)

[8.5 Study Halting Rules 7](#_Toc444769440)

[8.6 Safety Oversight 7](#_Toc444769441)

[9 CLINICAL MONITORING 7](#_Toc444769442)

[10 STATISTICAL CONSIDERATIONS 7](#_Toc444769443)

[10.1 Statistical and Analytical Plans 7](#_Toc444769444)

[10.2 Statistical Hypotheses 7](#_Toc444769445)

[10.3 Analysis Datasets 7](#_Toc444769446)

[10.4 Description of Statistical Methods 8](#_Toc444769447)

[10.4.1 General Approach 8](#_Toc444769448)

[10.4.2 Analysis of the Primary Efficacy Endpoint(s) 8](#_Toc444769449)

[10.4.3 Analysis of the Secondary Endpoint(s) 8](#_Toc444769450)

[10.4.4 Safety Analyses 8](#_Toc444769451)

[10.4.5 Adherence and Retention Analyses 8](#_Toc444769452)

[10.4.6 Baseline Descriptive Statistics 8](#_Toc444769453)

[10.4.7 Planned Interim Analyses 8](#_Toc444769454)

[10.4.9 Multiple Comparison/Multiplicity 8](#_Toc444769455)

[10.4.10 Tabulation of Individual Response Data 8](#_Toc444769456)

[10.4.11 Exploratory Analyses 8](#_Toc444769457)

[10.5 Sample Size 9](#_Toc444769458)

[10.6 Measures to Minimize Bias 9](#_Toc444769459)

[10.6.1 Enrollment/ Randomization/ Masking Procedures 9](#_Toc444769460)

[10.6.2 Evaluation of Success of Blinding 9](#_Toc444769461)

[10.6.3 Breaking the Study Blind/Participant Code 9](#_Toc444769462)

[11 SOURCE DOCUMENTS AND ACCESS TO SOURCE DATA/DOCUMENTS 9](#_Toc444769463)

[12 QUALITY ASSURANCE AND QUALITY CONTROL 9](#_Toc444769464)

[13 ETHICS/PROTECTION OF HUMAN SUBJECTS 9](#_Toc444769465)

[13.1 Ethical Standard 9](#_Toc444769466)

[13.2 Institutional Review Board 9](#_Toc444769467)

[13.3 Informed Consent Process 9](#_Toc444769468)

[13.3.1 Consent/assent and Other Informational Documents Provided to Participants 9](#_Toc444769469)

[13.3.2 Consent Procedures and Documentation 9](#_Toc444769470)

[13.4 Participant and data Confidentiality 10](#_Toc444769471)

[13.4.1 Research Use of Stored Human Samples,Specimens or Data 10](#_Toc444769472)

[13.5 Future Use of Stored Specimens 10](#_Toc444769473)

[14 DATA HANDLING AND RECORD KEEPING 10](#_Toc444769474)

[14.1 Data Collection and Management Responsibilities 10](#_Toc444769475)

[14.2 Study Records Retention 10](#_Toc444769476)

[14.3 Protocol Deviations 10](#_Toc444769477)

[14.4 Publication and Data Sharing Policy 10](#_Toc444769478)

[15 STUDY ADMINISTRATION 10](#_Toc444769479)

[15.1 Study Leadership 10](#_Toc444769480)

[16 CONFLICT OF INTEREST POLICY 10](#_Toc444769481)

[17 LITERATURE REFERENCES 10](#_Toc444769482)

[APPENDIX 11](#_Toc444769483)

#

# LIST OF ABBREVIATIONS

| AT | Adipose tissue |
| --- | --- |
| SAT | Subcutaneous adipose tissue |
| VAT | Visceral adipose tissue |
| MRI | Magnetic resonance imaging |
| RMR | Resting metabolic rate |
| PA | Physical activity |
| WC | Waist circumference |
| TG | Triglyceride |

# STATEMENT OF COMPLIANCE

The trial will be carried out in accordance with Good Clinical Practice (GCP) as required by the by the Medical Ethics Board and the Helsinki Committee of the Soroka University Medical Center.

All key personnel (all individuals responsible for the design and conduct of this trial) have completed Human Subjects Protection Training.

The PI will ensure that all staff members involved in the conduct of this study are informed about their
obligations in meeting the above commitments.

Principal Investigator: Dr. Ilan Shelef, MD

# PROTOCOL SUMMARY

| **Title:** | **Differential effect of weight-loss diet strategies on dynamics of abdominal and other fat depots and metabolic risk – the CENTRAL study: intervention trial** |
| --- | --- |
| **Précis:** | **Methods**: an estimate of 250 participants who have either a. central obesity or b. lean participants with poor dietary habits combined with hyper TG will be randomized by median VAT to the following groups: **Group 1:** low-fat diet. **Group 2 :** Low-carb /Mediterranean high MUFA and PUFA diet diet. Specific food items will be provided. After 6 months of intervention, the diet groups 1 and 2 will be re-randomized to continue with diet only or with combination of 4 hours/week aerobic and resistance gym exercise for the subsequent year, divided by 4 equal size groups of **Group A** (low-fat diet only), **Group A+** (low-fat diet +physical activity) , **Group B** (Low-carb/ high MUFA & PUFA diet only) and **Group B+** (Low-carb/ high MUFA & PUFA diet + physical activity). All groups will be guided for their dietary intervention by nutritional sessions. All the participants will be followed by 1.5-Tesla MRI imaging of fat and muscle, blood and urine biomarkers, clinical measurements (weight, waist circumference, blood pressure, energy expenditure) and electronic questionnaire of dietary, lifestyle and medical information. Furthermore, genetic tests will be performed following a genetic ethical committee approval. |
| **Objectives:** | To assess whether distinct lifestyle strategies can differentially affect specific body adipose pools.  ***Objective 1:***  To compare the effects of: low-carb/high-MUFA; and the currently recommended diet by the American Heart Association (low-fat diet) on the dynamics of body fat compesition (mainly VAT) during weight loss and regain-plateau phases by MRI with and without physical activity. ***Objective 2:***  To assess the effect of Low-carb/MED and Low- fat diets with or without PA on the dynamics of abdominal suncotenius intra-hepatic fat among participants with abdominal obesity or dyslipidemia. |
| **Endpoint** | Primary Endpoint: Body fat composition (changes in VAT). Important Secondary Endpoints: changes in abdominal subcutaneous fat sub-depots and hepatic fat. |
| **Population:** | Healthy adults with abdominal obesity/dyslipidemia. |
| **Phase:** | Phase 3 |
| **Number of Sites enrolling participants:** | Nuclear Research Center-Negev, Dimona, Israel  250 participants |
| **Description of Study Agent :** | Lifestyle (a low-fat (LF) or Mediterranean/low-carbohydrate (MED/LC+28g walnuts/day) diet with/without added moderate physical activity) intervention |
| **Study Duration:** | 42 months |
| **Participant Duration:** | 18 months  *Time (in months) it will take for each individual participant to complete all participant visits* |

# SCHEMATIC OF STUDY DESIGN

**Flow chart of the CENTRAL study**


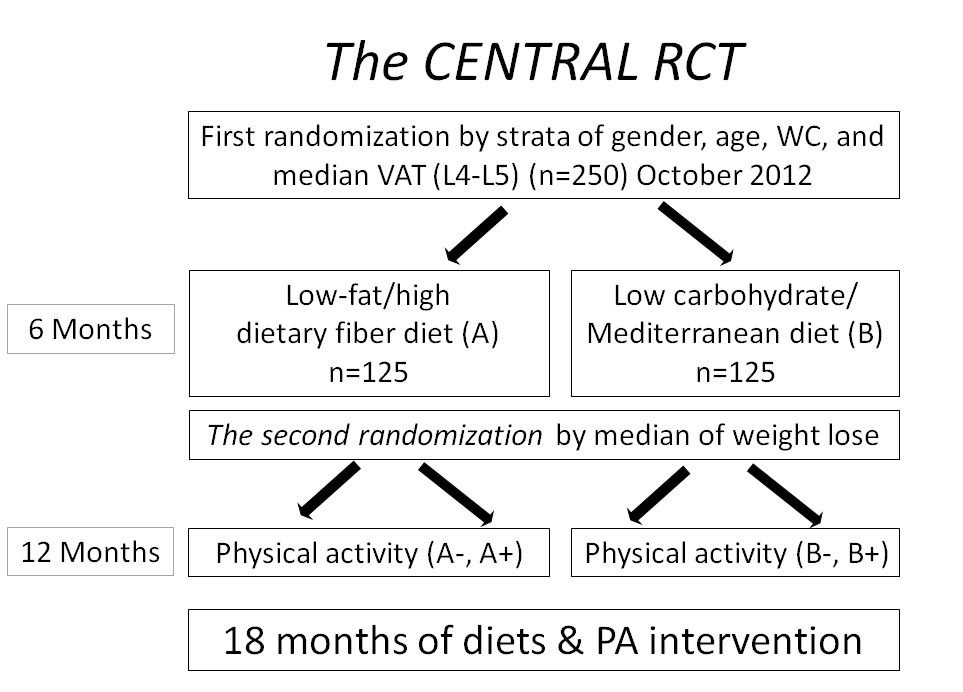


# 1 KEY ROLES

Principal Investigator: Dr. Ilan Shelef, MD, Soroka Medical Center

Sub-Investigators: Dr. Iris Shai, Ph.D., Ben-Gurion University of the Negev

Dr. Dan Schwarzfuchs, MD, Nuclear Research Center-Negev

Yftach Gepner, PhD student, Ben-Gurion University of the Negev

Clinical laboratory: The laboratory of the University of Leipzig, Germany.

# 2 INTRODUCTION: BACKGROUND INFORMATION AND SCIENTIFIC RATIONALE

## 2.1 Background Information

**Introduction:**

*Abdominal obesity signifies fat distribution associated with increased risk for obesity-related co-morbidity*. Obesity – the conversion of chronic surplus in calories into excessive accumulation of adipose tissue – is becoming a major threat to human health in the twenty-first century^1^. The association between obesity and cardio-metabolic risk does not follow a simple linear relationship^2^; Whereas some individuals have largely increased body mass index (BMI) with minimal health consequence^3^, others display a complex of metabolic, endocrine and vascular abnormalities with even mild increase in BMI beyond the accepted upper limit of 25 kg/m^2^. The reasons for this variability in the toll of excessive adipose tissue accumulation on health are largely unknown. Yet, *the anatomical locations in which adipose tissue accumulates* may be a significant factor^4,5^; As noted decades ago, fat accumulation in the lower part of the body, also called gynoid obesity, is rather benign and may even be protective against obesity-related morbidity. In contrast, upper body fat accumulation, particularly in the abdominal region, strongly associates with increased risk for diabetes and cardiovascular disease.  *Abdominal fat consists of several distinct fat depots*. SAT, represents fat accumulation outside the abdominal cavity ^6^. Within the abdominal cavity fat accumulates mainly around the internal organs- VAT and in the omentum. Among moderately obese healthy men large inter individual differences are observed for total adipose tissue (AT) volume (6.9-59.3 liters), SAT (6.3-49.8 liters), and VAT (0.5-8.5 liters)^7^. VAT represented 18.3% of the total AT. A distinct retroperitoneal fat also exists. Multiple studies now demonstrate that different fat depots are in fact quite distinct in their metabolic and endocrine functions (^2,5^ and **Table 1**). Firstly, while the venous drainage of subcutaneous and retroperitoneal fat is to the systemic circulation, visceral and omental fat drain into the portal system, thus, communicating directly with the liver by virtue of secreted products. Yet in addition, gene array analyses demonstrate significant differences between the fat depots in multiple developmental genes, suggesting intrinsic, genetically-encoded heterogeneity between fat tissues ^8^. These translate into important functional differences, including relatively increased sensitivity of intra-abdominal fat to lipolytic stimuli, and differential secretion of various fat-derived "adipokines". Rodent models suggest that in response to chronic excess in calories, intra-abdominal fat mainly responds by hypertrophy (and large adipocytes are thought to be more insulin resistant and "dysfunctional"), whereas subcutaneous fat tends to respond by hyperplasia^9^. In established obesity the cellular composition of adipose tissue in different depots is also distinct, with macrophages infiltrating to a larger degree the intra-abdominal versus the SAT^10, 11^. *Intriguingly, although increased waist circumference correlates with increased intra-abdominal fat mass, it may in fact represent predominant expansion of different depots in the abdomen.* In fact, some individuals exhibit a predominant accumulation of abdominal subcutaneous or of intra-abdominal fat^11, 12^, potentially resulting in comparable waist circumference. The distinction between persons with "predominant intra-abdominal (IA) adiposity" versus those with "predominant subcutaneous (SC) adiposity" relies on determining the SC/IA ratio based on imaging approaches^11^, as will be described in Section c.2. Intriguingly, when measured, it is the intra-abdominal fat mass which best correlated with glucose intolerance, insulin resistance, low HDL, high TG and hypertension^13, 14^. *Visceral fat is therefore suggested as a unique, pathogenic fat depot^15, 16^*. Yet, a *causative role* for intra-abdominal fat in the co-morbidity of obesity is not well established, and is still debated.

**Table 1: Differences between VAT and SAT (Reviewed in^2, 5, 17^)**

| **Difference** | **SAT** | **VAT** |
| --- | --- | --- |
| *Anatomic:* Venous drainage  *Morphology:* Adipocyte cell size  *Cell composition:* infiltrating macrophages | Systemic  ++  + | Portal  +  ++ |
| *Metabolic:* Glucose uptake, synthesis and storage rate of triglycerides | + | ++ |
| *Endocrine:*  Insulin-stimulation of glucose uptake and signaling  Anti-lipolytic action of insulin, or alpha-2 adrenergic agonists  Lipolysis in response to beta-adrenergic stimuli  11-β-HSD expression | +  ++  +  + | ++  +  ++  ++ |
| *Adipokines and secreted products:*  Leptin  Adiponectin  IL-6, IL-8, PAI-1, angiotensinogen | ++  +  + | +  ++  +++ |
| *Proliferantion:*  Tendency to proliferate of pre-adipocytes  Expression of PPAR-gamma and response to TZD | ++  ++ | +  + |
| *Stress pathways:*  Expression of JNK, p38MAPK, IKK, ERK  Phosphorylation (activation) of JNK and p38MAPK | +  + | ++  +++ |

*There are several comparable dietary strategies to induce weight loss, but their effect on specific abdominal fat depots is unclear*. The effectiveness for weight loss^18-20^, and superiority for cardiovascular benefits^21^ of low-fat diets components is debated. Recently we completed a 2-years intensive workplace-based Dietary Intervention Randomized Controlled Trial (DIRECT)^22^ among 322 participants, who were assigned to three intervention arms - Low-fat diet, Mediterranean diet (MED), and low-carbohydrate (low-carb) diet. Adherence was 95% at 1 year and 85% at 2 years. The results [see details in the preliminary (c.3.) and figures sections] suggest that whereas the low-carb diet was superior in improving lipid profile and weight loss, the Med diet (high in mono-unsaturated fat-MUFA) provided the largest decrease in circulating insulin and glucose levels. All three arms induced similar dynamics in weight, revealing a weight-loss phase (0-6 months) and a subsequent regain-plateau (6-24 months) phase (N Engl J Med 2008). Specific loss of VAT mass has mostly been shown in response to exercise training^23^. Gene expression of adiponectin in VAT and SAT is modified by physical training^24^. However, dietary strategy to preferentially promote loss of VAT is unknown. Several lines of evidence suggest differential roles of macronutrients in the *accumulation* of intra-abdominal (visceral) adipose tissue. VAT cells have a two-fold higher glucose uptake rate and de-novo lipogenesis capacity compared with SAT cells^25^. Glucose-stimulated insulin secretion may also have preferential effects on VAT, as insulin-stimulated glucose uptake and lipogenesis, and the stimulation of the expression of 11-β-HSD1 with resulting increase in active cortisol, are more evident in VAT, enhancing VAT accumulation^26^. Feeding rats with a high sucrose diet increase not only muscle insulin resistance, but also VAT mass^27^, at least partly by promoting cell hypertrophy^28,29^. In a 4-week intervention study among 22 human obese subjects, a larger decrease in VAT measured by computed tomography was observed in the low-carbohydrate diet group compared to the high-carbohydrate diet group^30^. *It is therefore possible that reducing carbohydrate load may preferentially decrease VAT accumulation*. In contrast, dietary fat explained only two percent of the variance in general adiposity, and was unrelated to VAT mass when measured in cross-sectional studies^31^. A recent study on 42 monkeys compared diets containing either monounsaturated fatty acids or an equivalent diet containing trans-fats for 6 years^32^. It was shown that trans-fat-fed monkeys gained significantly more weight, associated with increased intra-abdominal fat deposition and insulin resistance, compared to monkeys fed diet rich in monounsaturated fatty acids. In humans, in a cross-sectional study among 334 female twins there was no relationship between total dietary fat composition and adiposity^33^. However, in women at low genetic risk of abdominal obesity, subjects with higher polyunsaturated fat intakes had about 50% less central abdominal fat than subjects with lowest intake. Thus, a suggested preventive strategy for VAT accumulation may include limiting dietary carbohydrates and apparently, increasing monounsaturated fats^34^. Therefore, some evidence suggests that VAT accumulation may be linked to carbohydrate load in the diet, whereas the fat load (accept for trans-fatty acids) plays a minor, or even protective role (in the case of monounsaturated fat). *However, large-scale intervention trials assessing whether limiting carbohydrates and increasing monounsaturated fat can assist in inducing VAT loss beyond the weight loss induced by conventional hypocaloric diet are required to establish the relationship between dietary macronutrients composition, intraabdominal adiposity and insulin resistance.*

## 2.2 Rationale

Although accumulation of intra-abdominal fat may be pathogenic, it is unknown whether different diets improve metabolic state through differential effects on intra-abdominal versus subcutaneous-abdominal adipose tissue. While inducing similar global weight loss, low-carbohydrate/Mediterranean (LC/MED) diet strategy more favorably affects "pathogenic" abdominal fat depots when compared to the conventional low-fat diet (LF). These nutritional strategies initiated on top of exercise may manifest by differences in the maximal extent and/or dynamics of decrease in the mass of abdominal fat depots, and correspond to improvement in metabolic risk markers. For this, we propose an 18-month dietary and exercise intervention randomized controlled trial (the CENTRAL study). We hypothesize that differential dynamics in various fat depots: as SAT and VAT in response to two opposing dietary strategies mediate the beneficial metabolic effects during weight loss and regain phases.

Significance – The results of the present study will enhance current understanding on the mechanistic role of different abdominal fat depots (mainly VAT) in the pathogenesis of obesity. Particularly, it will increase current resolution in linking *dynamic* alterations in abdominal fat depots with various components of obesity-related metabolic dys-regulation. From a nutritional point of view, it will unravel the potential differential effect of macronutrients (carbs versus fat) in driving central adiposity. As for public health, the results will shed light on effective, health-promoting lifestyle intervention strategies, and whether they can be tailored individually based on abdominal fat distribution at baseline. This would be achieved by means of addressing the major limitations of currently available studies in the literature, by utilizing long-term intervention study with an expected high adherence rates.

## 2.3 Potential Risks and Benefits

### 2.3.1 Known Potential Risks

• Discomfort during MRI and RMR procedures.
• Dedication of time to workshops, filling out questionnaires and various follow-up tests.
• Discomfort during the blood draw.
• To the best of our knowledge, no risk to participants is expected.

### 2.3.2 Known Potential Benefits

• Participation in nutrition workshops with clinical dietitians.

• Free gym membership (for half of the participants).
• Imaging of fat tissue in the body. A radiologist will review the findings of the anatomical scan and create contact if further investigation is required.
• Receiving a detailed personal report summarizing all their measurements after the completion of the trial.

# 3 OBJECTIVES AND PURPOSE

***Objective 1:***  To assess the effect of Low-carb/MED and Low- fat diets with or without PA on the dynamics of body fat compesition (mainly VAT) during weight loss and regain-plateau phases by 1.5-Tesla MRI.

***Objective 2:***  To assess the effect of Low-carb/MED and Low- fat diets with or without PA on the dynamics of abdominal suncotenius intra-hepatic fat among participants with abdominal obesity or dyslipidemia.

# 4 STUDY DESIGN AND ENDPOINTS

## 4.1 Description of the Study Design

**Experimental design and methods**

*Global approach and study population:* The proposed 18-month intervention study will commence in one phase in a large research center workplace, located in Dimona in the south of Israel. The suitability of this setting is proven in several studies characterized by tight follow-up and/or interventions, which we performed in recent years^22,36-37^. Its advantages include an integral medical center, the possibility to perform most procedures (intervention and follow-up) within the workplace, and a secluded set of cafeterias amenable to dietary intervention Randomization- After performance of baseline measurements, the enrolled participants will be entered sequentially onto a list of random group assignments without stratification.

**Intervention**: In this a randomized clinical trial, participants will be randomized first for two diet groups: A. Low-fat diet; B. Low carbohydrate/Mediterranean diet, with 28g a day walnut supply, rich in polyunsaturated fatty acids (PUFA). All groups will be counseled by a clinical dietitian in dietary sessions that will delivered at work, 90 minutes each, every week during the first month of intervention, and then every month for the remainder of the trial. Diets will be equal-caloric for the entire study period. **Group A** – Low fat diet based on American Heart Association guidelines, will be guided to consume 30% of calories from fat, 10% of calories from saturated fat, and an intake of up to 300 mg of cholesterol per day. **Group B** – Low-carb/Mediterranean (high PUFA) diet is based on a combination and optimization of the Low-carb and Mediterranean diets tested in the DIRECT trial. Participants will be required to consume up to 20–40g of carbohydrates per day for the 2-month induction phase and immediately after religious holidays, with a gradual increase to a maximum of 120g per day to maintain their weight loss. Additionally, 28 g of fresh walnuts will be provided from the second month of intervention (see Table 2 for more details).

The second randomization will be performed after six months of the dietary intervention. The dietary groups will be further divided into PA (A+, B+) and non-PA (A-, B-) groups. The participants assigned to PA groups will get 12 months free supervise gym membership. The PA intervention will be mostly aerobic exercise training (80%) and resistance training three times a week, for 30 minutes in the first month, 50 minutes in the second to fifth months, and 80 minutes for months six to twelfth. The PA participants will be guided first in the gym with the support of the professional sport trainers. We will follow the PA participants online by electronic gym card recording their frequency of access and duration to the sport center and will call them frequently to encourage adherence.

***Intervention:*  Arm A:** the 2006 currently recommended diet by the American Heart Association (AHA) ^35^ (Low-fat) calorie restricted diet, a more revised statement since the AHA 2000; **Arm B:** guidelines^40^Low-carb/MED non-calorie restricted diet (n=125). Based on our preliminary results, low-carb and Mediterranean diets each showed beneficial effects on several biomarkers. Thus, here we suggest to utilize an optimized diet strategy which combines the beneficial components of low carb (low-carb) and Mediterranean (high monounsaturated) diets (**Table 2**). For both arms moderate physical activity will be equally introduced and monitored. The participants will be guided to exercise 150 min/week of moderate intensity, primarily in the form of initiating walking. This will be monitored by podometers that will be provided. The advantage of this strategy is that the podometer reflects both exercise thermogenesis (walking) as well as components of Non-exercise activity thermogensis (NEAT)^41^. The format and quality of the sessions will be kept constant to ensure equal treatment intensity, except for the specific instructions and paper material distributed for each diet strategy.

*Strategies to promote and verify adherence^22^: i. Food color and nutrition data labeling:* Lunch is considered the main daily meal in Israel. As the self-serve cafeteria in the workplace is the exclusive source of lunch for the participants, we will label each food item and nutritional data for the entire cafeteria styles. After analyzing the recipes and raw foods using the Israeli nutritional database^42^, we will display in each food-label: calories, grams of carbohydrates, fat, and saturated fat per portion size. We will further color-code the food item labels by setting specific criteria to component contents of each diet. Each dish will be labeled with a full circle (= "feel free to consume") or half-circle (= "consume in moderation") according to the diet arm, using two different colors. *ii.* The entire intervention and most measurements will be performed in the workplace, continuously monitored by the nurse study. Furthermore, structured motivation telephone calls and ongoing messaging via the intranet communication system will be held by specifically-trained dietitians.

The participants will be required to fill in electronic questionnaires at baseline, 6, and 18 months, which include: i. a validated food frequency questionnaire (FFQ) ^43^ that includes 127 food items and 3 portion sizes pictures for 17-selected food items. The nutritional data will be analyzed in the Israeli nutrition database ^42^. ii. Physical activity questionnaire: transformation to metabolic equivalents ^39^. Medical information will be updated by the workplace medical center’s physicians. A uniquely-developed electronic interface for submitting the questionnaires will ensure the completeness of data by popping-up missing lines.^22^

**Table 2: Dietary intervention strategies**

|  | **Arm B: Low-carbohydrate/high monounsaturated diet** | **Arm A (control, AHA recommendation): high-carbohydrate/ low-saturated**  **fat diet** |
| --- | --- | --- |
| Energy | Non-restricted | Restricted |
| Total fat | Liberal, expected: >40% | < 35% |
| Saturated fat | not specifically restricted | < 7-10% |
| Dietary cholesterol | not specifically restricted | < 300 mg |
| Trans fats | not recommended | not recommended |
| Carbohydrates | Restricted: < 40gr in the first 2 months (induction phase), then gradually increasing to a maximum of 80 grams to maintain achieved weight loss,  expected: <35% | Not restricted, whole grains are recommended, expected: >55% |
| Specific foods added | 45 gr virgin olive oil/day ( expected at least 20% monounsaturated fat)  25 gr/day nuts  2 fish /wk |  |

### 4.2.1 Primary Endpoint

Changes in body fat compestion (mainly VAT) following the intervention

### 4.2.2 Secondary Endpoints

Changes in abdominal subcutaneous and hepatic fat following the intervention

### 4.2.3 Exploratory Endpoints

# 5 STUDY ENROLLMENT AND WITHDRAWAL

## 5.1 Participant Inclusion Criteria

In order to be eligible to participate in this study, an individual must meet all of the following criteria:

• Either abdominal obesity [waist circumference >102cm (40 inches) for men and >88cm (35 inches) for women], or serum triglycerides (TG)>150mg/dL and high-density-lipoprotein cholesterol (HDL‑c) <40mg/dL for men and <50mg/dL for women.
• Provision of signed and dated informed consent form
• Stated willingness to comply with all study procedures and availability for the duration of the
study.

## 5.2 Participant Exclusion Criteria

An individual who meets any of the following criteria will be excluded from participation in this study:

• Serum creatinine≥2mg/dl

• Impaired liver function (≥3 fold the upper level of ALT and AST enzymes)

• Active cancer

• Pregnancy or lactation

• Being highly physically active (more than 3 hours/week) or inability to take part in PA

• Participation in another trial.

## 5.3 Strategies for Recruitment and Retention

Participants’ recruitment

The recruitment will be conducted among employees of the Nuclear Research Center, Dimona, Israel. The trial will be announced in the workplace and the potential participants will be contacted by the on-site clinic staff. The suitability of this setting is proven in several studies characterized by tight follow-up and/or interventions, which we performed in recent years^22^. Its advantages include an integral medical center, the possibility to perform most procedures within the workplace, and a secluded set of cafeterias amenable to dietary intervention.

Monitoring and Motivating Adherence

Adherence to the diet will be assessed by monitoring attendance of the nutritional sessions, and will be quantified via a self-administered validated electronic 127 item food-frequency questionnaires (FFQ) at baseline and after 6 and 18 months of intervention. By using electronic questionnaires, the completeness of the data was ensured by prompting the participants when a question was not answered or when an answer was not within a logical range. Adherence to the exercise intervention will be monitored frequently during the monthly group meetings and also quantified by calculating the METs/week using an electronic self-reported validated physical activity questionnaires at baseline and after 6 and 18 months. To motivate compliance, participants will be given a detailed individual report summarizing all their measurements after the completion of the trial. Participants who will not attend nutritional sessions or will decrease their gym attendance, as tracked online by the electronic gym entry monitor, will be contacted by telephone. Text messages will be sent to update participants and to motivate adherence to the diets on specific occasions (such as before and after holidays). Symptoms, adverse effects, quality of life, and medication usage will be measured at baseline and after 6 and 18 month, using validated electronic questionnaires^37, 42, 43^.

## 5.4 Participant Withdrawal or termination

5.4.1 Reasons for Withdrawal or Termination

Participants are free to withdraw from participation in the study at any time upon request.
An investigator may terminate participation in the study if any clinical adverse event (AE), laboratory abnormality, or other medical condition or situation occurs such that continued participation in the study would not be in the best interest of the participant

5.4.2 Handling of Participant Withdrawals or termination

The participants who withdraw or discontinue early will not be replaced.

## 5.5 Premature Termination or Suspension of Study

April 2014

# 6 STUDY AGENT

## 6.1 Study Agent(s) and Control Description

**Diet Intervention**

Both diets are aimed for a moderate, long-term, weight loss with restricted intake of *trans*-fats and refined carbohydrates, and increased intake of vegetables. Lunch, typically the main meal in this population, will be provided exclusively by the workplace cafeteria. A dietitian will be working closely with the kitchen staff to adjust the diets to the specific diet groups. The 18-month dietary intervention will include 90-minute nutritional session in the workplace with clinical dietitians every week during the first month of the intervention, and every month thereafter. Participants will be instructed to adhere to their specific diets during the entire week. To ensure that the intensity of treatment was equal between the two dietary intervention groups, the workshop format and the quality of the materials delivered during these sessions will be similar across the diet groups.

For the LF diet, the aim is to limit total fat intake to 30% of calories, with up to 10% of saturated fat, and no more than 300 mg of cholesterol per day, and to increase dietary fiber. Participants will be counseled to consume whole grains, vegetables, fruits, and legumes and to limit their consumption of additional fats, sweets, and high-fat snacks.

The MED/LC diet will combine the Mediterranean and low-carbohydrate diets described in our previous weight loss trial (the DIRECT trial^22^). The diet restricted carbohydrate intake to less than 40 g/day in the first two months (induction phase), and thereafter a gradual increase up to 70 g/day, and increased protein and fat intake, according to the MED diet. The MED/LC diet will be rich in vegetables and legumes and low in red meat, with poultry and fish replacing beef and lamb. This group will be also provided 28 g of walnuts/day [160 Kcal/84% fat, mostly PUFA (omega-3 α-linolenic acid)] starting from the third month, after the very low carbohydrate diet during the first two months.

**Physical Activity Intervention**

Starting after the first 6 months of dietary intervention, participants who will be randomized to added PA will receive a free supervised gym membership for the following 12 months. The intervention will include monthly 60-minute educational workshops, and training group sessions at the gym, directed by a certified fitness instructor, who will be blinded to the assigned diets of the participants. The exercise program will include three sessions/week of mostly aerobic training. In the first month participants will start with low intensity of aerobic training at 65% maximum heart rate for 20 minutes and 10 minutes of resistance training. Exercise will gradually increase to 45 minutes of aerobic training at 80% of maximum heart rate and 15 minutes of resistance training. The resistance training will be increased from one set of weights with 60% of the maximum weight up to two sets with 80% of the maximum weight and will include: leg extension, leg curl, elbow flexion, triceps extension, lateral pull-down, lower back extension and bent leg sit-ups.

### 6.1.1 Acquisition

NA

### 6.1.2 Formulation, Appearance, Packaging, and Labeling

NA

### 6.1.3 Product Storage and Stability

NA

### 6.1.4 Preparation

NA

### 6.1.5 Dosing and Administration

NA

### 6.1.6 Route of Administration

NA

### 6.1.7 Starting Dose and Dose Escalation Schedule

NA

### 6.1.8 Dose Adjustments/Modifications/Delays

NA

### 6.1.9 Duration of Therapy

18-month intervention

### 6.1.10 Tracking of Dose

NA

### 6.1.11 Device Specific Considerations

NA

## 6.2 Study agent Accountability Procedures

# 7 STUDY PROCEDURES AND SCHEDULE

## 7.1 Study Procedures/Evaluations

### 7.1.1 Study specific procedures

**Magnetic Resonance Imaging (MRI)**

To assess body fat depots/deposits, we will perform a 45-minute 1.5-Tesla MRI (Ingenia 3.0 T, Philips Healthcare, Best, the Netherlands) scans at baseline and after 18 months. In a sub-set of, randomly selected, 50% of the participants, we will perform MRI measurement after 6 months of intervention. The scanner utilize a 3D modified DIXON (mDIXON) imaging technique without gaps (2mm thickness and 2mm of spacing), fast-low-angle shot (FLASH) sequence with a multi-echo two-excitation pulse sequence for phase-sensitive encoding of fat and water signals (TR,3.6ms; TE1,1.19ms; TE2,2.3ms; FOV 520×440×80mm; 2×1.4×1mm voxel size). Four images of the phantoms will be generated, including in-phase, out-phase, fat and water phase. A breath-hold technique will be used to avoid motion artifacts when the chest and abdomen will be scanned. In all simultaneous fat depots quantifications and comparisons, observers will be blinded to time point and group treatment. All fat depots will be assessed by one or two raters. To validate our mDIXON quantification of fat ratio, we will test five tubes filled with 0%, 25%, 50%, 75% and 100% of oil to observe a linear relation.

***Abdominal fat depots:*** We will quantify abdominal fat using the MATLAB-based semi-automatic software. We will calculate mean VAT and SAT from three axial slices: L5-S1, L4-L5 and L2-L3. Quantification of the fat mass regions will include the area of each fat type and its proportion (percentage) of the total area of all fat types.

***Hepatic fat content:*** We will quantify the percentage of hepatic fat using PRIDE software from Philips Medical Systems. We will calculate mean percentage from four 2D slices (3cm intervals divided into quarters) by utilizing the region of interest (ROI) approach, which is based on measurements of tissue densities (fat/fat+water) using the Fat Ratio Calculation.

***Resting metabolic Rate (RMR)*** We will mesure RMR by indirect calorimetry (QUARK REE by COSMED, Rome, Italy) in which the quantity of oxygen consumed and carbon dioxide produced are measured and converted into kcal using the Weir equation.

**Clinical measurements**

Height will be measured at baseline, to the nearest millimeter by using a standard wall-mounted stadiometer. WC will be measured at baseline, after 6 months (prior to the second randomization), and at 18 months, to the nearest millimeter with an anthropometric measuring tape; the measurement will be made half-way between the last rib and the iliac crest. Body weight will be measured monthly without shoes to the nearest 0.1kg. Fasting blood samples will be taken at baseline and at 18 months, at 8:00am, and will be stored at ‑80°C. Fasting plasma glucose (FPG) will be measured by Roche GLUC 3 (hexokinase method). Plasma insulin will be measured with an enzyme immunometric assay [Immulite automated analyzer, Diagnostic Products, coefficient of variation (CV)=2.5%]. Serum total cholesterol (CV=1.3%), high-density-lipoprotein cholesterol (HDL-c), low-density-lipoprotein (LDL) cholesterol, and triglycerides (CV=2.1%) will be determined enzymatically with a Cobas 6000 automatic analyzer (Roche). Plasma leptin levels will be assessed by ELISA (Mediagnost, CV=2.4%). All biochemical analyses will be performed at the laboratories of the University of Leipzig, Germany.

Changes in resting metabolic rate (RMR) will be performed, using a handheld indirect calorimeter^52^.

### 7.1.2 Standard of care study procedures

NA

## 7.2 Laboratory Procedures/Evaluations

NA

### 7.2.1 Clinical Laboratory Evaluations

### 7.2.2 Other Assays or Procedures

### 7.2.3 Specimen Preparation, Handling, and Storage

### 7.2.4 Specimen Shipment

All the samples will be transported with appropriate bio-material shipping.

## 7.3 Study Schedule

### 7.3.1 Screening

Please refer to Section 7.3.7, Schedule of Events.

### 7.3.2 Enrollment/Baseline

Please refer to Section 7.3.7, Schedule of Events.

### 7.3.3 Follow-up

Please refer to Section 7.3.7, Schedule of Events.

### 7.3.4 Final Study Visit

Please refer to Section 7.3.7, Schedule of Events.

### 7.3.5 Early Termination Visit

NA

### 7.3.6 UNSCHEDULED VISIT

NA

### 7.3.7 Schedule of Events Table

**Table 3: Design of the intervention; the CENTRAL study**

| Year | 2012 | | | | 2013 | | | | | | | | | | | | 2014 | | |
| --- | --- | --- | --- | --- | --- | --- | --- | --- | --- | --- | --- | --- | --- | --- | --- | --- | --- | --- | --- |
| Month of intervention  Event | 0 | 1 | 2 | 3 | 4 | 5 | 6 | 7 | 8 | 9 | 10 | 11 | 12 | 13 | 14 | 15 | 16 | 17 | 18 |
| Dietary sessions |  | ++ | + | + | + | + | + | + | + | + | + | + | + | + | + | + | + | + | + |
| Physical activity intervention – gym membership provided |  |  |  |  |  |  |  | ++ | + | + | + | + | + | + | + | + | + | + | + |
| Whole body MRI | + |  |  |  |  |  | + |  |  |  |  |  |  |  |  |  |  |  | + |
| REE – Indirect calorimetry | + |  |  |  |  |  | + |  |  |  |  |  |  |  |  |  |  |  | + |
| Blood draw and urine samples (fasting) | + |  |  |  |  |  | + |  |  |  |  |  |  |  |  |  |  |  | + |
| Anthropometric measurements (WC, BP) | + |  |  |  |  |  | + |  |  |  |  |  |  |  |  |  |  |  | + |
| Weight measurement | + | + | + | + | + | + | + | + | + | + | + | + | + | + | + | + | + | + | + |
| Electronic questionnaires (FFQ, medical, symptoms, life-style, PA) | + |  |  |  |  |  | + |  |  |  |  |  |  |  |  |  |  |  | + |
| Physician follow-up (including reporting updates in drug usage, safety) |  |  |  |  |  |  | + |  |  |  |  |  | + |  |  |  |  |  | + |

## 7.4 Justification for Sensitive Procedures

NA

## 7.5 Concomitant Medications, Treatments, and Procedures

The medical clinic of the workplace will continue with the routine screening test and treatment

### 7.5.1 Precautionary Medications, Treatments, and Procedures

## 7.6 Prohibited Medications, Treatments, and Procedures

The medical clinic of the workplace will continue with the routine screening test and treatment

## 7.7 Prophylactic Medications, Treatments, and Procedures

The medical clinic of the workplace will continue with the routine screening test and treatment

## 7.8 Rescue Medications, Treatments, and Procedures

The medical clinic of the workplace will continue with the routine screening test and treatment

## 7.9 Participant Access to Study Agent At Study Closure

Participants will be able to be in contact with all investigators during the study and after the study will be complete

# 8 ASSESSMENT OF SAFETY

## 8.1 Specification of Safety Parameters

### 8.1.1 Definition of Adverse Events (AE)

Adverse event means any untoward medical occurrence associated with the use of an intervention in
humans, whether or not considered intervention-related (21 CFR 312.32 (a)).

### 8.1.2 Definition of Serious Adverse Events (SAE)

Serious adverse event or serious suspected adverse reaction. An AE or suspected adverse reaction is
considered "serious" if, in the view of either the investigator or sponsor, it results in any of the following
outcomes: death, a life-threatening adverse event, inpatient hospitalization or prolongation of existing
hospitalization, a persistent or significant incapacity or substantial disruption of the ability to conduct
normal life functions, or a congenital anomaly/birth defect. Important medical events that may not
result in death, be life-threatening, or require hospitalization may be considered serious when, based
upon appropriate medical judgment, they may jeopardize the patient or subject and may require
medical or surgical intervention to prevent one of the outcomes listed in this definition.

### 8.1.3 Definition of Unanticipated Problems (UP)

OHRP considers unanticipated problems involving risks to participants or others to include, in general,
any incident, experience, or outcome that meets **all** of the following criteria:
• Unexpected in terms of nature, severity, or frequency given (a) the research procedures that are
described in the protocol-related documents, such as the IRB-approved research protocol and
informed consent document; and (b) the characteristics of the participant population being
studied;
• Related or possibly related to participation in the research (“possibly related” means there is a
reasonable possibility that the incident, experience, or outcome may have been caused by the
procedures involved in the research); and
• Suggests that the research places participants or others at a greater risk of harm (including
physical, psychological, economic, or social harm) than was previously known or recognized.
This study will use the OHRP definition of UP.

## 8.2 Classification of an Adverse Event

### 8.2.1 Severity of Event

The following guidelines will be used to describe severity:
• **Mild** – Events require minimal or no treatment and do not interfere with the participant’s daily
activities.
• **Moderate** – Events result in a low level of inconvenience or concern with the therapeutic
measures. Moderate events may cause some interference with functioning.
• **Severe** – Events interrupt a participant’s usual daily activity and may require systemic drug
therapy or other treatment. Severe events are usually potentially life-threatening or
incapacitating.

### 8.2.2 Relationship to Study Agent

For all collected AEs, the clinician who examines and evaluates the participant will determine the AE’s
causality based on temporal relationship and his/her clinical judgment. The degree of certainty about
causality will be graded using the categories below.
• **Definitely Related** – There is clear evidence to suggest a causal relationship, and other possible
contributing factors can be ruled out. The clinical event, including an abnormal laboratory test
result, occurs in a plausible time relationship to drug administration and cannot be explained by
concurrent disease or other drugs or chemicals. The response to withdrawal of the drug
(dechallenge) should be clinically plausible. The event must be pharmacologically or
phenomenologically definitive, with use of a satisfactory rechallenge procedure if necessary.
• **Probably Related** – There is evidence to suggest a causal relationship, and the influence of other
factors is unlikely. The clinical event, including an abnormal laboratory test result, occurs within
a reasonable time after administration of the drug, is unlikely to be attributed to concurrent
disease or other drugs or chemicals, and follows a clinically reasonable response on withdrawal
(dechallenge). Rechallenge information is not required to fulfill this definition.
• **Possibly Related** – There is some evidence to suggest a causal relationship (e.g., the event
occurred within a reasonable time after administration of the trial medication). However, other
factors may have contributed to the event (e.g., the participant’s clinical condition, other
concomitant events). Although an AE may rate only as “possibly related” soon after discovery, it
can be flagged as requiring more information and later be upgraded to “probably related” or
“definitely related,” as appropriate.
• **Unlikely to be related** – A clinical event, including an abnormal laboratory test result, whose
temporal relationship to drug administration makes a causal relationship improbable (e.g., the
event did not occur within a reasonable time after administration of the trial medication) and in
which other drugs or chemicals or underlying disease provides plausible explanations (e.g., the
participant’s clinical condition, other concomitant treatments).
• **Not Related** – The AE is completely independent of study drug administration, and/or evidence
exists that the event is definitely related to another etiology. There must be an alternative,
definitive etiology documented by the clinician.] <Insert text>

### 8.2.3 Expectedness

NA

## 8.3 Time Period and Frequency for Event Assessment and Follow-Up

The occurrence of an AE or SAE may come to the attention of study personnel during study visits and
interviews of a study participant presenting for medical care, or upon review by a study monitor.
All AEs including local and systemic reactions not meeting the criteria for SAEs will be captured on the
appropriate CRF. Information to be collected includes event description, time of onset, clinician’s
assessment of severity, relationship to study product (assessed only by those with the training and
authority to make a diagnosis), and time of resolution/stabilization of the event. All AEs occurring while
on study must be documented appropriately regardless of relationship. All AEs will be followed to
adequate resolution.
Any medical condition that is present at the time that the participant is screened will be considered as
baseline and not reported as an AE. However, if the study participant’s condition deteriorates at any
time during the study, it will be recorded as an AE.
Changes in the severity of an AE will be documented to allow an assessment of the duration of the event
at each level of severity to be performed. AEs characterized as intermittent require documentation of
onset and duration of each episode.
The PI will record all reportable events with start dates occurring any time after informed consent is
obtained until 7 (for non-serious AEs) or 30 days (for SAEs) after the last day of study participation. At
each study visit, the investigator will inquire about the occurrence of AE/SAEs since the last visit. Events
will be followed for outcome information until resolution or stabilization.

## 8.4 Reporting Procedures

### 8.4.1 Adverse Event Reporting

Notifications regarding AEs will receive Dr. Ilan Shelef, MD, Soroka Medical Center (PI) and Dr. Dan Schwarzfuchs, MD, Nuclear Research Center-Negev (the head of the integral medical center in Nuclear Research Center).

### 8.4.2 Serious Adverse Event Reporting

### 8.4.3 Unanticipated Problem Reporting

NA

### 8.4.4 Events of Special Interest

### 8.4.5 Reporting of Pregnancy

Women participants will be required to report their pregnancy to Dr. Dan Schwarzfuchs, MD, Nuclear Research Center-Negev (the head of the integral medical center in the workplace) and the intervention will be discontinued.

## 8.5 Study Halting Rules

NA

## 8.6 Safety Oversight

# 9 CLINICAL MONITORING

The medical clinic of the workplace will continue with the routine screening test and treatment

# 10 STATISTICAL CONSIDERATIONS

## 10.1 Statistical and Analytical Plans

The overall primary outcome is the change in body fat distribution, and the main specific endpoint is VAT, with a priori hypothesis that VAT, specifically, could be differentially and significantly altered by lifestyle intervention strategies. Secondary outcomes will be abdominal subcutaneous fat sub-depot and hepatic fat.

## 10.2 Statistical Hypotheses

We hypothesize that differential dynamics in various fat depots: as SAT and VAT in response to two opposing dietary strategies mediate the beneficial metabolic effects during weight loss and regain phases.

## 10.3 Analysis Datasets

We will check the assumptions for all the statistical analyses to ensure that none were violated. We will perform intention-to-treat analyses, including all the participants, by imputing the missing observations for adipose tissues for the individuals with missing data at 18-months by the multiple imputation technique, with the following predictors: age, gender, baseline weight/ BMI and waist circumference at the end of the intervntion. For missing data of body weight, we will use the last observation carried forward.

## 10.4 Description of Statistical Methods

### 10.4.1 General Approach

Results will be presented as means ± SD unless otherwise stated. Analysis of variance with a covariance (ANCOVA) test will be used in order to assess adherence to the interventions (attendance of the group sessions) and differences in changes nutrient intakes between the diet strategies. General linear models will be used to compare the trajectories of body weight and WC across the four intervention groups. We will use multivariate two-way ANCOVA and compare type of diet and physical activity as fixed factors (2*2 factorial design) to test changes in specific fat depots, using mean of differences and 95% CIs for each intervention group over time. Optional interactions between diet and exercise will be assessed by the ANCOVA models. To assess the effect of the combination of diet and PA, general linear models will be used with LFPA– as the reference. All models will be adjusted for age, sex, baseline abdominal obesity and 18-month weight change. Changes in the various fat depots and deposits will be evaluated both as absolute and relative changes. The association between changes in abdominal fat and intra-hepatic fat, and changes in levels of blood biomarkers will be tested using multivariate linear regression models, adjusted for age, sex, weight change, intervention group and baseline abdominal fat status (for the abdominal fat models) or baseline intra-hepatic fat (for the hepatic model). Homeostasis-model-assessment-of insulin-resistance (HOMA-IR) will be calculated according to the following equation: insulin (U/ml)×fasting glucose(mmol/liter) ÷22.5. Statistical analysis will be performed with IBM SPSS (version 23). All *P*-values will be two-sided, and *P*<0.05 will be considered statistically significant.

### 10.4.2 Analysis of the Primary Efficacy Endpoint(s)

The overall primary outcome is the change in body fat distribution, and the main specific endpoint is VAT.

### 10.4.3 Analysis of the Secondary Endpoint(s)

Secondary outcomes will be abdominal subcutaneous fat sub-depot and hepatic fat.

### 10.4.4 Safety Analyses

NA

### 10.4.5 Adherence and Retention Analyses

Adherence to the diet and exercise will be assesse monthly by monitoring attendance to the sessions, and also by questionnaires (FFQ and PA) at baseline and after 6 and 18 months.

### 10.4.6 Baseline Descriptive Statistics

At baseline we will preforme ANOVA and t-test to demonestrat diffrences between the study groups

### 10.4.7 Planned Interim Analyses

NA

#### 10.4.7.1 Safety Review

#### 10.4.7.2 Efficacy Review

#### 10.4.8 Additional Sub-Group Analyses

Sensitivity analyses within the stratum of men only will be test

### 10.4.9 Multiple Comparison/Multiplicity

### 10.4.10 Tabulation of Individual Response Data

### 10.4.11 Exploratory Analyses

## 10.5 Sample Size

Sample size was estimated based on findings from a previous 14-week intervention study, in which 33 postmenopausal obese women (57 yr, 92 kg, 36% body fat) were randomized to one of three interventions: diet alone, exercise alone, and diet and exercise group. We observed a significant relative change in VAT of 12.8% (P<0.05). Thus, the minimum detectable effect after 18 months for the primary VAT between the intervention groups was estimated as 3.57cm^2^, and for an alpha=5% and power=80%, 250 participants were required (calculated using Winpepi software).

## 10.6 Measures to Minimize Bias

### 10.6.1 Enrollment/ Randomization/ Masking Procedures

Participants will be randomly assigned to one of two equal-caloric diets for the entire study period: a low-fat diet (n=125) or a Mediterranean/low-carbohydrate diet (n=125). After 6 months of dietary intervention, each diet group will be further randomized into added PA groups (LF^PA+^, MED/LC^PA+^) or no added PA groups (LF^PA–^, MED/LC^PA–^) for the last 12 months of intervention. The randomization will be performed with an allocation ratio of 1:1 to the two treatment groups, within strata of baseline VAT area for the first randomization, and of address of residence (to ensure similar geographical distance from the gym) in the second randomization (in blocks of two). Participants will be randomized after all had been recruited, in one phase, after their strata characteristics will be defined.

The participants, who will be randomized in the same time point, will be aware of the intervention to which they will be assigned (open-label). Study investigators will be blinded to the intervention group identities.

### 10.6.2 Evaluation of Success of Blinding

Double Blind (Care Provider, Investigator, Outcomes Assessor)

### 10.6.3 Breaking the Study Blind/Participant Code

The code will be blind until the end of the study.

# 11 SOURCE DOCUMENTS AND ACCESS TO SOURCE DATA/DOCUMENTS

All documents, include the singed inform consent will be hold in a lock room in the Radiolegy department in Soroka Medical Center.

# 12 QUALITY ASSURANCE AND QUALITY CONTROL

# 13 ETHICS/PROTECTION OF HUMAN SUBJECTS

## 13.1 Ethical Standard

The PI will ensure that this study is conducted in full conformity with the Declaration of Helsinki.

## 13.2 Institutional Review Board

The protocol, informed consent form, recruitment materials, and all participant materials were submitted and approved by the IRB (Medical Ethics Board and the Helsinki Committee of the Soroka University Medical Center, Israel). Any amendment to the protocol will be submitted for review and approval by the IRB before the changes are implemented to the study. All changes to the consent form will be IRB approved; a determination will be made regarding whether previously consented participants need to be re-consented.

## 13.3 Informed Consent Process

### 13.3.1 Consent/assent and Other Informational Documents Provided to Participants

Consent form describing in detail the intervention, study procedures, and risks are given to the
participant and written documentation of informed consent will be obtained prior to performing any study procedure.

### 13.3.2 Consent Procedures and Documentation

Informed consent is a process that will be initiated prior to the individual’s agreeing to participate in the
study and continues throughout the individual’s study participation. Extensive discussion of risks and
possible benefits of participation will be provided to the participants. Consent forms were IRB-approved and the participant will be asked to read and review the document. The investigator will explain the research study to the participant and answer any questions that may arise. All participants will receive a verbal explanation in terms suited to their comprehension of the purposes, procedures, and potential risks of the study and of their rights as research participants. Participants will have the opportunity to carefully review the written consent form and ask questions prior to signing. The participants will have the opportunity to discuss the study with their surrogates or think about it prior to agreeing to participate. The participant will sign the informed consent document prior to any procedures being done specifically for the study. The participants may withdraw consent at any time throughout the course of the trial. A copy of the informed consent document will be given to the participants for their records. The rights and welfare of the participants will be protected by emphasizing to them that the quality of their medical care will not be adversely affected if they decline to participate in this study.

## 13.4 Participant and data Confidentiality

To protect the participant confidentiality, immediately with the recruitment, each participant will be assigned a serial number. No personal identification will appear on any test or and sample, which will be marked only with a serial number. The list with participants' personal data and their serial number will be kept locked by the PI of the study. All the acquired data will be kept confidential and will be accessible only to the authorized staff.

Participant confidentiality is strictly held in trust by the participating investigators, their staff, and any additional research-involved personnel. This confidentiality is extended to cover testing of biological samples and genetic tests in addition to the clinical information relating to participants. Therefore, documentation, data, and all other information generated will be held in strict confidence. The study participant’s contact information will be securely stored at Soroka University Medical Center for internal use during the study. At the end of the study, all records will continue to be kept in a secure location for as long a period as dictated by local IRB and Institutional regulations.

Study participant research data, which is for purposes of statistical analysis and scientific reporting, will
be transmitted to and stored at the Ben-Gurion University of the Negev. This will not include the participant’s contact or identifying information. Rather, individual participants and their research data
will be identified by a unique study identification number. The study data entry and study management
systems used by clinical sites and by Ben-Gurion University of the Negev research staff will be
secured and password protected.

### 13.4.1 Research Use of Stored Human Samples,Specimens or Data

All the biological samples will be aliquoted and kept in the freezers of the nutrition center, which are accessible only to an authorized staff. At the end of the study, according to the participants’ consent, the samples collected under this protocol will be kept for future studies, for which we will separately submit the required documents and additional informed consent forms to IRB to obtain the proper approval (e.g. genetic sub-study). Data will be kept in password-protected computers. Only investigators will have access to the samples and data.

## 13.5 Future Use of Stored Specimens

With the participant’s approval and as was approved by local IR, coded biological samples will be
stored at the nutrition center of BGU. These samples could be used for further research.

After the study is completed, coded, archived data will be transmitted to and stored at the
BGU Data Repository, under the supervision of Prof. Shai, for use by other researchers including those outside of the study. For any future studies, a separate request for the study approval will be submitted to the Medical Ethics Board and the Helsinki Committee of the Soroka University Medical Center (IRB). To protect the participants’ confidentiality, all the stored specimens and/or data will be coded. Future studies may include performing genetic testing.

During the conduct of the study, an individual participant can choose to withdraw consent to have
biological specimens stored for future research.

# 14 DATA HANDLING AND RECORD KEEPING

## 14.1 Data Collection and Management Responsibilities

Data collection is the responsibility of the clinical trial staff at the site under the supervision of the site
PI. The investigator is responsible for ensuring the accuracy, completeness, legibility, and timeliness of
the data reported.
All source documents should be completed in a neat, legible manner to ensure accurate interpretation
of data. When making changes or corrections, cross out the original entry with a single line, and initial and date the change. DO NOT ERASE, OVERWRITE, OR USE CORRECTION FLUID OR TAPE ON THE ORIGINAL.

Copies of the electronic CRF (eCRF) will be provided for use as source documents and maintained for
recording data for each participant enrolled in the study. Data reported in the eCRF derived from source
documents should be consistent with the source documents or the discrepancies should be explained
and captured in a progress note and maintained in the participant’s official electronic study record.
Clinical data (including AEs, concomitant medications, and expected adverse reactions data) and clinical
laboratory data will be entered into the designated data storage system, which will include password protection and internal quality checks, such as automatic range checks, to identify data that appear inconsistent, incomplete, or inaccurate. Clinical data will be entered directly from the source documents.

## 14.2 Study Records Retention

Study documents will be retaine for 2 years after the final research report submission.

## 14.3 Protocol Deviations

A protocol deviation is any noncompliance with the clinical trial protocol, GCP, or MOP requirements.
The noncompliance may be either on the part of the participant, the investigator, or the study site staff.
As a result of deviations, corrective actions are to be developed by the site and implemented promptly.

The PI and the study staff is responsible for knowing and adhering to the IRB requirements.

## 14.4 Publication and Data Sharing Policy

At the end of the study, the PI will be responsible to make results of the research available to the research community and public at large. The trial was registered in a public trials registry: ClinicalTrials.gov, number: NCT01530724.

# 15 STUDY ADMINISTRATION

## 15.1 Study Leadership

Dr. Ilan Shelef, MD, Soroka Medical Center and Dr. Iris Shai, Ph.D., Ben-Gurion University of the Negev.

# 16 CONFLICT OF INTEREST POLICY

Authors have no conflict of interest to disclose.

# 17 LITERATURE REFERENCES

1. Kopelman PG. Obesity as a medical problem. Nature 2000;404(6778):635-43.

2. Yang X, Smith U. Adipose tissue distribution and risk of metabolic disease: does thiazolidinedione-induced adiposet issue redistribution provide a clue to the answer? Diabetologia 2007;50:1127-39.

3. Karelis AD, St-Pierre DH, Conus F, Rabasa-Lhoret R, Poehlman ET. Metabolic and body composition factors in subgroups of obesity: what do we know? J Clin Endocrinol Metab 2004;89(6):2569-75.

4. Kissebah AH, Krakower GR. Regional adiposity and morbidity. Physiol Rev 1994;74(4):761-811.

5. Wajchenberg BL. Subcutaneous and visceral adipose tissue: their relation to the metabolic syndrome. Endocr Rev 2000;21:697-738.

6. Smith SR, Lovejoy JC, Greenway F, Ryan D, deJonge L, de la Bretonne J, Volafova J, Bray GA. Contributions of total body fat, abdominal subcutaneous adipose tissue compartments, and visceral adipose tissue to the metabolic complications of obesity. Metabolism 2001;50(4):425-35.

7. Ross R, Leger L, Morris D, de Guise J, Guardo R. Quantification of adipose tissue by MRI: relationship with anthropometric variables. J Appl Physiol 1992;72(2):787-95.

8. Gesta S, Bluher M, Yamamoto Y, Norris AW, Berndt J, Kralisch S, Boucher J, Lewis C, Kahn CR. Evidence for a role of developmental genes in the origin of obesity and body fat distribution. Proc Natl Acad Sci U S A 2006;103(17):6676-81.

9. DiGirolamo M, Fine JB, Tagra K, Rossmanith R. Qualitative regional differences in adipose tissue growth and cellularity in male Wistar rats fed ad libitum. Am J Physiol 1998;274(5 Pt 2):R1460-7.

10. Cancello R, Tordjman J, Poitou C, Guilhem G, Bouillot JL, Hugol D, Coussieu C, Basdevant A, Hen AB, Bedossa P, Guerre-Millo M, Clement K. Increased infiltration of macrophages in omental adipose tissue is associated with marked hepatic lesions in morbid human obesity. Diabetes 2006;55(6):1554-61.

11. Harman-Boehm I, Bluher M, Redel H, Sion-Vardy N, Ovadia S, Avinoach E, Shai I, Kloting N, Stumvoll M, Bashan N, Rudich A. Macrophage infiltration into omental versus subcutaneous fat across different populations: effect of regional adiposity and the co-morbidities of obesity. J Clin Endocrionl Metab 2007;92:2240-7.

12. Despres JP, Lemieux I. Abdominal obesity and metabolic syndrome. Nature 2006;444(7121):881-7.

13. Despres JP, Moorjani S, Lupien PJ, Tremblay A, Nadeau A, Bouchard C. Regional distribution of body fat, plasma lipoproteins, and cardiovascular disease. Arteriosclerosis 1990;10(4):497-511.

14. Pouliot MC, Despres JP, Nadeau A, Tremblay A, Moorjani S, Lupien PJ, Theriault G, Bouchard C. Associations between regional body fat distribution, fasting plasma free fatty acid levels and glucose tolerance in premenopausal women. Int J Obes 1990;14(4):293-302.

15. Fox CS, Massaro JM, Hoffmann U, Pou KM, Maurovich-Horvat P, Liu CY, Vasan RS, Murabito JM, Meigs JB, Cupples LA, D'Agostino RB, Sr., O'Donnell CJ. Abdominal visceral and subcutaneous adipose tissue compartments: association with metabolic risk factors in the Framingham Heart Study. Circulation 2007;116(1):39-48.

16. Pou KM, Massaro JM, Hoffmann U, Vasan RS, Maurovich-Horvat P, Larson MG, Keaney JF, Jr., Meigs JB, Lipinska I, Kathiresan S, Murabito JM, O'Donnell CJ, Benjamin EJ, Fox CS. Visceral and subcutaneous adipose tissue volumes are cross-sectionally related to markers of inflammation and oxidative stress: the Framingham Heart Study. Circulation 2007;116(11):1234-41.

17. Rudich A, Kanety H, Bashan N. Adipose stress-sensing kinases: linking obesity to malfunction. Trends Endocrinol Metab 2007;18(8):291-9.

18. Willett WC. Dietary fat plays a major role in obesity: no. Obes Rev 2002;3(2):59-68.

19. Willett WC. The Mediterranean diet: science and practice. Public Health Nutr 2006;9(1A):105-10.

20. Hu FB. Protein, body weight, and cardiovascular health. Am J Clin Nutr 2005;82(1 Suppl):242S-7S.

21. Stampfer MJ, Hu FB, Manson JE, Rimm EB, Willett WC. Primary prevention of coronary heart disease in women through diet and lifestyle. N Engl J Med 2000;343(1):16-22.

22. Shai I, Schwarzfuchs D, Henkin Y, Shahar D, Witkow S, Greenberg I, et al. Weight Loss with Low-Carbohydrate, Mediterranean, or Low-Fat Diets. N Engl J Med 2008,;359(3):229-41.

23. Giannopoulou I, Ploutz-Snyder LL, Carhart R, Weinstock RS, Fernhall B, Goulopoulou S, Kanaley JA. Exercise is required for visceral fat loss in postmenopausal women with type 2 diabetes. J Clin Endocrinol Metab 2005;90(3):1511-8.

24. Bluher M, Williams CJ, Kloting N, Hsi A, Ruschke K, Oberbach A, Fasshauer M, Berndt J, Schon MR, Wolk A, Stumvoll M, Mantzoros CS. Gene expression of adiponectin receptors in human visceral and subcutaneous adipose tissue is related to insulin resistance and metabolic parameters and is altered in response to physical training. Diabetes Care 2007.

25. Lundgren M, Buren J, Ruge T, Myrnas T, Eriksson JW. Glucocorticoids down-regulate glucose uptake capacity and insulin-signaling proteins in omental but not subcutaneous human adipocytes. J Clin Endocrinol Metab 2004;89(6):2989-97.

26. Bujalska IJ, Kumar S, Stewart PM. Does central obesity reflect "Cushing's disease of the omentum"? Lancet 1997;349(9060):1210-3.

27. Kim JY, Nolte LA, Hansen PA, Han DH, Kawanaka K, Holloszy JO. Insulin resistance of muscle glucose transport in male and female rats fed a high-sucrose diet. Am J Physiol 1999;276(3 Pt 2):R665-72.

28. Keno Y, Matsuzawa Y, Tokunaga K, Fujioka S, Kawamoto T, Kobatake T, Tarui S. High sucrose diet increases visceral fat accumulation in VMH-lesioned obese rats. Int J Obes 1991;15(3):205-11.

29. Lewis GF, Carpentier A, Adeli K, Giacca A. Disordered fat storage and mobilization in the pathogenesis of insulin resistance and type 2 diabetes. Endocr Rev 2002;23(2):201-29.

30. Miyashita Y, Koide N, Ohtsuka M, Ozaki H, Itoh Y, Oyama T, Uetake T, Ariga K, Shirai K. Beneficial effect of low carbohydrate in low calorie diets on visceral fat reduction in type 2 diabetic patients with obesity. Diabetes Res Clin Pract 2004;65(3):235-41.

31. Larson DE, Hunter GR, Williams MJ, Kekes-Szabo T, Nyikos I, Goran MI. Dietary fat in relation to body fat and intraabdominal adipose tissue: a cross-sectional analysis. Am J Clin Nutr 1996;64(5):677-84.

32. Kavanagh K, Jones KL, Sawyer J, Kelley K, Carr JJ, Wagner JD, Rudel LL. Trans fat diet induces abdominal obesity and changes in insulin sensitivity in monkeys. Obesity (Silver Spring) 2007;15(7):1675-84.

33. Greenfield JR, Samaras K, Jenkins AB, Kelly PJ, Spector TD, Campbell LV. Moderate alcohol consumption, dietary fat composition, and abdominal obesity in women: evidence for gene-environment interaction. J Clin Endocrinol Metab 2003;88(11):5381-6.

34. Freedland ES. Role of a critical visceral adipose tissue threshold (CVATT) in metabolic syndrome: implications for controlling dietary carbohydrates: a review. Nutr Metab (Lond) 2004;1(1):12.

35. [American Heart Association Nutrition Committee](http://www.ncbi.nlm.nih.gov/sites/entrez?Db=pubmed&Cmd=Search&Term=%22American%20Heart%20Association%20Nutrition%20Committee%22%5BCorporate%20Author%5D&itool=EntrezSystem2.PEntrez.Pubmed.Pubmed_ResultsPanel.Pubmed_DiscoveryPanel.Pubmed_RVAbstractPlus), [Lichtenstein AH](http://www.ncbi.nlm.nih.gov/sites/entrez?Db=pubmed&Cmd=Search&Term=%22Lichtenstein%20AH%22%5BAuthor%5D&itool=EntrezSystem2.PEntrez.Pubmed.Pubmed_ResultsPanel.Pubmed_DiscoveryPanel.Pubmed_RVAbstractPlus), [Appel LJ](http://www.ncbi.nlm.nih.gov/sites/entrez?Db=pubmed&Cmd=Search&Term=%22Appel%20LJ%22%5BAuthor%5D&itool=EntrezSystem2.PEntrez.Pubmed.Pubmed_ResultsPanel.Pubmed_DiscoveryPanel.Pubmed_RVAbstractPlus), [Brands M](http://www.ncbi.nlm.nih.gov/sites/entrez?Db=pubmed&Cmd=Search&Term=%22Brands%20M%22%5BAuthor%5D&itool=EntrezSystem2.PEntrez.Pubmed.Pubmed_ResultsPanel.Pubmed_DiscoveryPanel.Pubmed_RVAbstractPlus), [Carnethon M](http://www.ncbi.nlm.nih.gov/sites/entrez?Db=pubmed&Cmd=Search&Term=%22Carnethon%20M%22%5BAuthor%5D&itool=EntrezSystem2.PEntrez.Pubmed.Pubmed_ResultsPanel.Pubmed_DiscoveryPanel.Pubmed_RVAbstractPlus), [Daniels S](http://www.ncbi.nlm.nih.gov/sites/entrez?Db=pubmed&Cmd=Search&Term=%22Daniels%20S%22%5BAuthor%5D&itool=EntrezSystem2.PEntrez.Pubmed.Pubmed_ResultsPanel.Pubmed_DiscoveryPanel.Pubmed_RVAbstractPlus), [Franch HA](http://www.ncbi.nlm.nih.gov/sites/entrez?Db=pubmed&Cmd=Search&Term=%22Franch%20HA%22%5BAuthor%5D&itool=EntrezSystem2.PEntrez.Pubmed.Pubmed_ResultsPanel.Pubmed_DiscoveryPanel.Pubmed_RVAbstractPlus), et al. Diet and lifestyle recommendations revision 2006: a scientific statement from the American Heart Association Nutrition Committee. [Circulation.](javascript:AL_get(this,%20'jour',%20'Circulation.');) 2006;114(1):82-96.

36. Henkin Y, Shai I, Zuk R, Brickner D, Zuilli I, Neumann L, Shany S. Dietary treatment of hypercholesterolemia: do dietitians do it better? A randomized, controlled trial. Am J Med 2000;109(7):549-55.

37. Shai I, Rosner BA, Shahar DR, Vardi H, Azrad AB, Kanfi A, Schwarzfuchs D, Fraser D. Dietary evaluation and attenuation of relative risk: multiple comparisons between blood and urinary biomarkers, food frequency, and 24-hour recall questionnaires: the DEARR study. J Nutr 2005;135(3):573-9.

38. Thamer C, Machann J, Stefan N, Haap M, Schafer S, Brenner S, Kantartzis K, Claussen C, Schick F, Haring H, Fritsche A. Hwfigh visceral fat mass and high liver fat are associated with resistance to lifestyle intervention. Obesity (Silver Spring) 2007;15(2):531-8.

39. Ainsworth BE, Haskell WL, Whitt MC, Irwin ML, Swartz AM, Strath SJ, O'Brien WL, Bassett DR, Jr., Schmitz KH, Emplaincourt PO, Jacobs DR, Jr., Leon AS. Compendium of physical activities: an update of activity codes and MET intensities. Med Sci Sports Exerc 2000;32(9 Suppl):S498-504.

40. Krauss RM, Eckel RH, Howard B, Appel LJ, Daniels SR, Deckelbaum RJ, Erdman JW, Jr., Kris-Etherton P, Goldberg IJ, Kotchen TA, Lichtenstein AH, Mitch WE, Mullis R, Robinson K, Wylie-Rosett J, St Jeor S, Suttie J, Tribble DL, Bazzarre TL. AHA Dietary Guidelines: revision 2000: A statement for healthcare professionals from the Nutrition Committee of the American Heart Association. Circulation 2000;102(18):2284-99.

41. [Levine JA](http://www.ncbi.nlm.nih.gov/sites/entrez?Db=pubmed&Cmd=Search&Term=%22Levine%20JA%22%5BAuthor%5D&itool=EntrezSystem2.PEntrez.Pubmed.Pubmed_ResultsPanel.Pubmed_DiscoveryPanel.Pubmed_RVAbstractPlus), [Kotz CM](http://www.ncbi.nlm.nih.gov/sites/entrez?Db=pubmed&Cmd=Search&Term=%22Kotz%20CM%22%5BAuthor%5D&itool=EntrezSystem2.PEntrez.Pubmed.Pubmed_ResultsPanel.Pubmed_DiscoveryPanel.Pubmed_RVAbstractPlus). NEAT--non-exercise activity thermogenesis--egocentric & geocentric environmental factors vs. biological regulation. [Acta Physiol Scand.](javascript:AL_get(this,%20'jour',%20'Acta%20Physiol%20Scand.');) 2005;184(4):309-18.

42. Shai I, Vardi H, Shahar DR, Azrad AB, Fraser D. Adaptation of international nutrition databases and data-entry system tools to a specific population. Public Health Nutr 2003;6(4):401-6.

43. Shai I, Shahar DR, Vardi H, Fraser D. Selection of food items for inclusion in a newly developed food-frequency questionnaire. Public Health Nutr 2004;7(6):745-9.

44. Bonekamp S, Ghosh P, Crawford S, Solga SF, Horska A, Brancati FL, Diehl AM, Smith S, Clark JM. Quantitative comparison and evaluation of software packages for assessment of abdominal adipose tissue distribution by magnetic resonance imaging. Int J Obes (Lond) 2007.

45. Kuk JL, Church TS, Blair SN, Ross R. Does measurement site for visceral and abdominal subcutaneous adipose tissue alter associations with the metabolic syndrome? Diabetes Care 2006;29(3):679-84.

46. Abate N, Garg A, Coleman R, Grundy SM, Peshock RM. Prediction of total subcutaneous abdominal, intraperitoneal, and retroperitoneal adipose tissue masses in men by a single axial magnetic resonance imaging slice. Am J Clin Nutr 1997;65(2):403-8.

47. Liou TH, Chan WP, Pan LC, Lin PW, Chou P, Chen CH. Fully automated large-scale assessment of visceral and subcutaneous abdominal adipose tissue by magnetic resonance imaging. Int J Obes (Lond) 2006;30(5):844-52.

48. Gray DS, Fujioka K, Colletti PM, Kim H, Devine W, Cuyegkeng T, Pappas T. Magnetic-resonance imaging used for determining fat distribution in obesity and diabetes. Am J Clin Nutr 1991;54(4):623-7.

49. Machann J, Thamer C, Schnoedt B, Haap M, Haring HU, Claussen CD, Stumvoll M, Fritsche A, Schick F. Standardized assessment of whole body adipose tissue topography by MRI. J Magn Reson Imaging 2005;21(4):455-62.

50. Positano V, Gastaldelli A, Sironi AM, Santarelli MF, Lombardi M, Landini L. An accurate and robust method for unsupervised assessment of abdominal fat by MRI. J Magn Reson Imaging 2004;20(4):684-9.

51. Szczepaniak el al. Measurement of intracellular triglyceride stores by 1H spectroscopy: validation in vivo. *Am J Physiol Endocrinol Metab (1999; 276:977-989.*

52. [I[McDoniel SO](http://www.ncbi.nlm.nih.gov/sites/entrez?Db=pubmed&Cmd=Search&Term=%22McDoniel%20SO%22%5BAuthor%5D&itool=EntrezSystem2.PEntrez.Pubmed.Pubmed_ResultsPanel.Pubmed_DiscoveryPanel.Pubmed_RVAbstractPlus). Systematic review on use of a handheld indirect calorimeter to assess energy needs in adults and children. nt J Sport Nutr Exerc Metab.](javascript:AL_get(this,%20'jour',%20'Int%20J%20Sport%20Nutr%20Exerc%20Metab.');) 2007;17(5):491-500.

53. Tomkins O, Shelef I, Kaizerman I, Eliushin A, Afawi Z, Misk A, Gidon M, Cohen A, Zumsteg D, Friedman A. Blood-Brain Barrier Disruption in Post- Traumatic Epilepsy. J Neurol Neurosurg Psych 2007;Accepted.

54. Al-Kwifi O, Shelef I, Farb RI, Stainsby J, Wright GA. High-resolution imaging of the intracranial arterial and venous systems following a single contrast injection. J Magn Reson Imaging 2006;24(2):267-73.

55. Ifergane G, Shelef I, Buskila D. Migraine and fibromyalgia developing after a pontine haemorrhage. Cephalalgia 2007;27(2):191.

# APPENDIX

| Version | Date | Significant Revisions |
| --- | --- | --- |
|  |  |  |
|  |  |  |
